# Association of OPG, TNF-α, and IL-1B Gene Variants With Periodontitis in a South African Population

**DOI:** 10.1016/j.identj.2026.109606

**Published:** 2026-05-04

**Authors:** Salma Kabbashi, Ndonwi Elvis Ngwa, Haly Holmes, Yvonne Prince, Manogari Chetty

**Affiliations:** a Department of Craniofacial Biology, Pathology, & Radiology, Faculty of Dentistry, University of the Western Cape, Cape Town, South Africa; b SAMRC/CPUT/Cardiometabolic Health Research Unit, Department of Biomedical Sciences, Faculty of Health & Wellness Sciences, Cape Peninsula University of Technology, Cape Town, South Africa; c Department of Oral Medicine & Periodontology, Faculty of Dentistry, University of the Western Cape, Cape Town, South Africa

**Keywords:** Periodontitis, Interleukin-1beta, Osteoprotegerin, Polymorphism, Tumour Necrosis Factor-alpha, South Africa

## Abstract

**Aim:**

This study aimed to investigate candidate single-nucleotide polymorphisms linked to periodontitis susceptibility in a Western Cape cohort, providing insights into population-specific host genetic factors.

**Materials and Methods:**

This observational case-control study recruited a total of 150 South African participants. Saliva samples were genotyped using the OpenArray QuantStudio 12K Flex qPCR System. Genotype and allele frequencies were assessed for Hardy–Weinberg equilibrium and tested for associations with clinical variables and disease status using Chi-square tests and logistic regression adjusted for age, gender, and smoking.

**Results:**

A total of 24 SNPs were analysed. Several genotypes showed suggestive associations with periodontitis risk prior to multiple testing correction. The OPG + 1181 CG, RANKL RL2 AG, and IL-17A + 197 GG genotypes were linked to higher plaque score. The TNF-α -238 GG genotype was associated with lower bleeding score and reduced disease risk (OR = 0.157, 95% CI: 0.039-0.638, *P* = .010), while IL-1B -511 GG genotype corresponded with a reduced disease risk (adjusted OR = 0.216, 95% CI: 0.054-0.867, *P* = .031). Conversely, OPG + 1181 CC was related to increased disease risk under multiple models (adjusted OR = 20.42, 95% CI: 1.95-213.9, *P* = .012). These associations lost significance after correction for multiple testing. Ethnicity-based subgroup analysis revealed differences in genotype distribution, while smoking status showed no effect.

**Conclusion:**

Genetic variants may influence periodontitis susceptibility, underscoring the importance of population-specific risk profiling and the need for replication in larger cohorts to support targeted diagnostics in resource-limited settings.

## Introduction

Periodontitis is a multifactorial inflammatory disease that arises from a complex interaction between the oral microbiome, mucosal barrier, host immune response, and environmental influences in susceptible individuals.^1^ The disease is initiated by dental biofilm accumulation, which triggers an inflammatory host response. This response elicits both protective and destructive effects on the periodontium.^2^ Susceptibility to periodontitis is further modulated by environmental risk factors such as poor oral hygiene, smoking, and diet which acts in the presence of a favourable genetic background.^3^

Genetic variation in host response to microbial challenge modulates individual susceptibility to periodontitis. Polymorphisms in genes regulating immune and inflammatory pathways have been implicated in the development and progression of periodontitis.^4^ Importantly, genetic variants distribution differs across populations, with significant ethnic variation in allele frequencies.^5^

The Global Burden of Disease Study 2021 estimates that severe periodontitis affects over 1 billion people globally, placing it among the most widespread chronic inflammatory conditions worldwide.^6^ Its burden is unequally distributed, with the highest prevalence reported in low- and middle-income countries (LMICs).^7^ Systematic reviews and WHO reports show that Sub-Saharan Africa bears a disproportionate burden of severe periodontitis.^7^^,^^8^

Nonetheless, African populations remain severely underrepresented in periodontitis genetic research.^9^ Achieving the Sustainable Development Goals (SDG) by 2030 in countries such as the Republic of South Africa (RSA) requires strengthened capacity to anticipate and manage health risks.^10^ Given the RSA’s complex population structure and widespread socioeconomic barriers to dental care, investigating genetic susceptibility to periodontitis is essential. Population-specific studies can reveal risk alleles unique to African populations, guiding targeted/precision management and supporting resource allocation for underserved communities.

This study aimed to investigate 24 candidate single nucleotide polymorphisms (SNPs) associated with periodontitis susceptibility in a South African (SA) population from the Western Cape. The investigated variants include loci within the *IL-1 gene* cluster (IL-1A, IL-1B, IL-1RN), other cytokine genes (*TNF-α, IL-4, IL-6, IL-10, IL-17A, IFN-γ*), and genes involved in innate immune and tissue-destructive pathways (*TLR4, MMP8*), as well as those regulating bone remodelling (*RANK, RANKL, OPG*), representing some of the most biologically relevant and frequently investigated genetic targets in periodontitis research.^11^ Despite extensive investigation in predominantly non-African populations,^5^^,^^12^, ^13^, ^14^, ^15^ reported associations remain inconsistent and population-specific, highlighting the need for replication in underrepresented African cohorts. This study represents the first replication of this set of candidate genetic variants across a multi-ethnic SA cohort, contributing to a more inclusive understanding of periodontitis.

## Materials and methods

### Study design and population

Ethical approval was obtained from the Biomedical Research Ethics Committee (BMREC) of University of the Western Cape (UWC), reference number BM20/10/9. This case-control study included 150 individuals: 75 periodontitis patients and 75 periodontally healthy controls. Classification of periodontal status followed the 2017 case definitions and guidelines of the American Academy of Periodontology (AAP) and the European Federation of Periodontology (EFP).^16^ Participants were recruited and data collected at the Tygerberg Oral Health Centre, University of the Western Cape, Cape Town, South Africa**,** between August 2021 and February 2023**.** Cases were drawn from the Department of Oral Medicine and Periodontology, while controls were primarily drawn from Orthodontics and Prosthodontics Departments in Tygerberg Oral Health Centre at UWC (Figure S1). The use of dental-department controls ensured comparable access to care and reduced socioeconomic and recall bias between groups.

Eligible participants were SA adults aged 18 years or older, having a minimum of 10 natural teeth. Initial screening was performed using the Basic Periodontal Examination (BPE), with scores of 3 or 4 indicating potential periodontal disease.^17^ Comprehensive periodontal assessments were subsequently conducted under optimal clinical conditions by the primary investigator, who was calibrated by a consultant periodontist to ensure diagnostic consistency.

Exclusion criteria included: individuals with diabetes mellitus, current tobacco users (≥ 10 times per day).^18^ Additional exclusions encompassed those with systemic diseases affecting the periodontium, participants with gingivitis, used antiseptic or anti-inflammatory drugs for more than 1 week in the preceding 3 months, received antibiotics or periodontal therapy in the past 3 months, or were pregnant, lactating, or completely edentulous.

Demographic and personal data, including age, gender, and self-reported ethnicity, were collected through structured interviews. Ethnicity was considered during analysis to account for potential population stratification within the SA cohort. All participants received study information sheets, and written informed consent was obtained in one of the 3 major languages spoken in RSA prior to participation.

Clinical periodontal parameters were recorded as full mouth measurement of probable pocket depth (PPD) and clinical attachment loss (CAL) at 6 sites per tooth (mesio-buccal, disto-buccal, mesio-lingual, disto-lingual, buccal, and lingual), except the third molars and radices. Full-mouth bleeding score (FMBS), full-mouth plaque score (FMPS), and mobility were also documented. Measurements were obtained using a standard William’s periodontal probe (no. PQW; Hu-Friedy, Chicago, IL, USA) and rounded to the nearest millimetre, with the aid of a mouth mirror and headlamp. The primary investigator demonstrated high intra-examiner reliability (κ > 0.81), ensuring consistency of clinical measurements across all participants. Panoramic radiographs, including orthopantomograms and intraoral views, were used as adjunctive diagnostic tools.

### Saliva sample collection

Unstimulated whole saliva was collected by passive drooling into a sterile Oragene G-500 tube (DNA Genotek Inc; RRID:SCR_027611), following the manufacturer's instructions. Participants were instructed to rinse their mouths and refrain from eating or drinking for at least 30 minutes prior to collection. Approximately 2 mL of saliva was provided after rubbing the tongue around the mouth for 15 seconds to enhance cell yield.^19^ A negative control kit was used to rule out clinician-induced contamination. Samples were stored at -20 °C until DNA extraction.

### Genetic analysis

#### DNA extraction

Saliva samples were shipped on ice to the Centre for Proteomic and Genomic Research (RRID:SCR_017158), for genetic analysis. Genomic DNA was extracted using the QIAamp DNA Blood Mini Kit (QIAGEN, RRID:SCR_008539, Cat.#51126) on the Qiagen QIAcube Classic (RRID:SCR_018618) platform, following the manufacturer’s protocol. DNA concentration and purity were measured using a Thermo Scientific NanoDrop 8000 Spectrophotometer (RRID:SCR_018600), samples ≥ 50 ng/μL and A260/280 = 1.7-1.9 were retained for analysis. DNA integrity was further assessed by agarose gel electrophoresis using E-Gel 48 1% Agarose Gels (Thermo Fisher Scientific, RRID:SCR_008452, Cat.#G800801), following the manufacturer’s instructions.

#### SNP genotyping

Genotyping was performed using custom TaqMan OpenArray plates on the Applied Biosystems QuantStudio 12K Flex RealTime PCR System (RRID:SCR_021098). Primer and probe assays for 22 SNPs were selected using Thermo Fisher’s assay search tool, while 2 SNPs (rs1800630 and rs2430561) were designed using the Custom TaqMan Assay Design Tool (Thermo Fisher Scientific, RRID:SCR_008452).

#### Investigated SNPs

A total of 24 SNPs across 12 candidate genes were selected for genotyping (Table S1), based on prior associations with periodontitis in other populations, allowing potential replication in the SA cohort, and on their involvement in key biological pathways relevant to disease pathophysiology.^20^ Only SNPs with genotype call rates > 95%were included in the final analysis.

### Population stratification

Population structure was evaluated using STRUCTURE (RRID:SCR_017637) under the admixture model with correlated allele frequencies, without prior population information. The analysis was run with a burn-in of 10,000 iterations followed by 20,000 MCMC repetitions, assuming K = 2 populations. STRUCTURE was used to assess underlying genetic substructure and admixture within the cohort, taking into account the candidate-gene design and the limited number of loci. The inferred ancestry proportions (Q values) indicated substantial admixture across the cohort; therefore, no individuals were excluded based on admixture status. STRUCTURE results were interpreted descriptively and were not used to derive ancestry covariates. Population heterogeneity was addressed analytically by conducting ethnicity-based subgroup analyses.

### Power calculation

A conservative approach was adopted, recruiting as many eligible participants as possible. The final sample comprised 150 individuals (75 cases, 75 controls), consistent with minimum sizes reported in similar studies.^21^ Post-hoc sensitivity analysis in G*Power (RRID:SCR_013726) (logistic regression, α = 0.00217 after Bonferroni correction, N = 150, case: control ratio = 1:1, R² = 0.02) showed that the study had 80% power to detect odds ratios of approximately OR ≥ 3.3 (for risk alleles with minor allele frequency (MAF) ≈ 0.30).

### Statistical analysis

All statistical analyses were performed using IBM SPSS Statistics (RRID:SCR_016479). Continuous variables were expressed as mean ± standard deviation (SD) and categorical variables as frequencies and percentages. Group differences were evaluated using the Student’s t-test or one-way ANOVA, as appropriate. Hardy–Weinberg equilibrium (HWE) was assessed for each SNP using the Chi-square test in GenAlEx (RRID:SCR_021102), and only SNPs in HWE were retained for association analyses. Genotype distributions were compared across subgroups (smoking status, ethnicity) using Chi-square tests, while associations between SNPs and clinical parameters were examined by one-way ANOVA and their relationship with periodontitis by logistic regression under additive, dominant, and recessive models, adjusted for confounders: age (modelled as continuous variable), sex, and smoking.

Given the candidate-gene design and the limited number of predefined SNPs, multiple testing was controlled using the Bonferroni correction, a conservative approach selected to minimise type I error in confirmatory association testing,^22^ and only results meeting the corrected threshold were considered statistically significant. Analyses were conducted at a 95% confidence level (*P* < .05).^23^

## Results

### Demographics and clinical parameters

The mean age of periodontitis cases (46.19±13.5 years) was nearly double that of controls (24.68±8.73 years), a difference that was statistically significant (*P* < .001) (Table 1). Age was therefore treated as a potential confounder and included as a covariate in all multivariable association analyses. There was no significant difference in gender distribution between the 2 groups. Participants represented all 4 SA ethnic groups, with the mixed-ancestry group, officially termed South African Coloured (SAC), being the most prevalent among both cases and controls. The Asian/Indian group was least represented among cases, and the Caucasian group among controls. Smoking was significantly more common in the periodontitis group (*P* = .003), with 35.6% current smokers, 16.4% former smokers, and 48.0% non-smokers, compared to 17.8%, 6.9%, and 75.3%, respectively, in controls. The periodontitis group showed significantly higher FMPS, FMBS, PPD, and CAL values, and a lower mean number of present teeth (all *P* < .001) (Table 1).

**Table 1:** Demographics and full mouth clinical parameters of periodontitis cases and periodontally healthy controls of the study population.

Parameters		Cases (n = 75)	Controls (n = 75)	*P*-value
Age in years (mean ± SD)		46.19±13.25	24.68±8.73	< .001*
Number of present teeth (mean ± SD)		23.15±5.84	29.2±3.07	< .001*
FMPS (mean ± SD)		40.5**±** 34.5	9.3**±** 5.7	< .001*
FMBS (mean ± SD)		56.77±34.34	3.31±2.99	< .001*
PPD (mean ± SD)		3.63±0.79	2.44±0.35	< .001*
CAL (mean ± SD)		4.07±0.98	1.90±2.09	< .001*
Gender n (%)	Female	46 (61.3)	51 (68.0)	.393
	Male	29 (38.7)	24 (32.0)	
Ethnicityn (%)	Caucasian	19 (25.3)	14 (19.2)	.003*
	African	11 (14.7)	18 (24.7)	
	SAC	42 (56.0)	26 (35.6)	
	Asian/Indian	3 (4.0)	15 (20.5)	
Smoking n (%)	Never smokers	35 (48.0)	55 (75.3)	.003*
	Former smokers	12 (16.4)	5 (6.9)	
	Current smokers	26 (35.6)	13 (17.8)	

CAL, clinical attachment loss; FMBS, bleeding on probing; FMPS, plaque index; N, number; P, probability; PD, pocket depth; SAC, South African Coloured; SD, standard deviation.

⁎ *P* < .05 indicates statistical significance.

**Figure 1:**
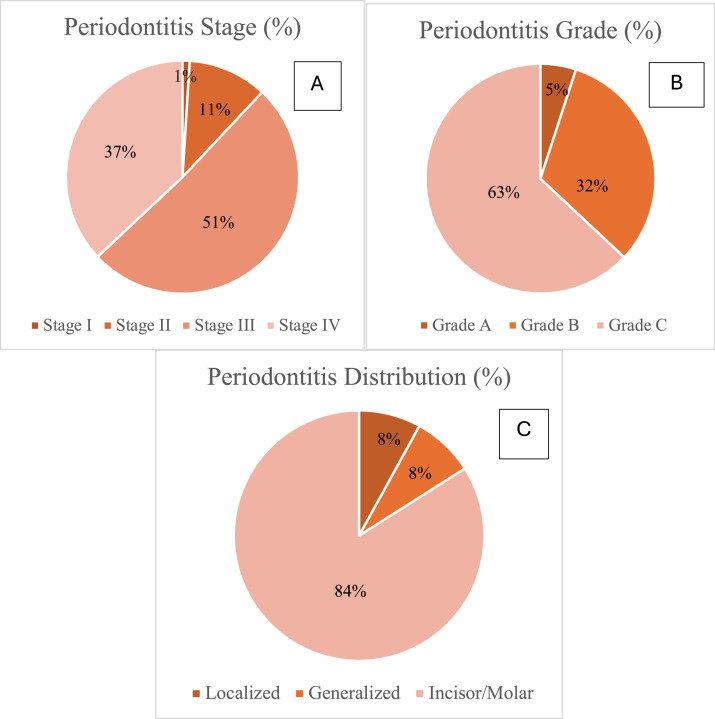
Periodontitis classification based on staging and grading systems. (A) Percentage of different periodontitis stages among cases group. (B) Percentage of different grades of periodontitis among case group participants. (C) Different distribution patterns of periodontitis among the cases*.*

Among periodontitis cases, stage III was the most prevalent (50.7%), while stage I was least common (1.3%). The majority of cases were classified as grade B (63.0%), followed by grade C (32.0%) and grade A (5.0%). The generalized distribution of periodontitis dominated, affecting 84% of cases, with other patterns each accounting for 8% (Figure 1). All diagnostic categories were represented, with Generalized Stage IV Grade B being the most frequent (25.3%). All controls were diagnosed with gingival and periodontal health.

### SNPs genotyping

All 24 SNPs were successfully genotyped, though some rare genotypes; IL-1RN + 2018 CC, TNF-α −238 AA, TLR-4 + 896 GG, and + 1196 TT, were absent in both cases and controls. All SNPs conformed to HWE, except IL-6 −572 (rs1800796), which deviated in controls (*P* = .027) and cases (*P* = .007) and was excluded from further analysis. The minor alleles were the same in both cases and controls for most SNPs. However, for IL-1B -511 (rs16944) the minor allele was G in cases and A in controls. There were no significant differences in the genotype and allele distribution of any of the studied SNPs between cases and controls (*P* > .05) (Table S2).

#### Periodontitis clinical parameters

Carriers of the GG genotype in TNF-α −238 (rs361525) had significantly lower FMBS than GA carriers (*P* = .043). For OPG + 1181 (rs2073618), significant differences in FMPS were observed across genotypes (*P* = .007), with CG carriers showing the highest mean FMPS. In RANKL RL2 (rs2277438), FMPS was highest in AG carriers (*P* = .038). For IL-17A + 197 (rs2275913), FMPS was significantly higher in GG carriers (*P* = .012). Significant results are summarized in Table 2, while non-significant findings are in Table S3.

**Table 2:** Association between SNP genotypes and clinical characteristics of periodontitis.

SNP genotype	Clinical parameters			
	FMPS (mean ± SD)	FMBS (mean ± SD)	PD (mean ± SD)	CAL (mean ± SD)
dbSNP ID				
	rs361525			
G/G (n = 128)	24.14±28.09	27.84±35.27	2.99±0.83	2.87±2.03
G/A (n = 22)	29.32±35.43	42.86±39.59	3.27±0.98	3.66±1.25
*P*-value	.854	.043*	.238	.401
	rs2073618			
C/C (n = 74)	20.57±26.33	30.58±36.88	3.01±0.79	3.00±1.72
C/G (n = 58)	34.12±33.58	33.90±36.83	3.13±0.99	3.03±2.31
G/G (n = 17)	12.82±14.44	16.29±29.04	2.79±0.54	2.79±1.75
*P*-value	.007*	.281	.461	.231
	rs2277438			
A/A (n = 108)	20.97±25.89	27.93±35.76	3.04±0.91	3.09±1.75
A/G (n = 37)	35.57±34.70	38.92±38.07	3.06±0.69	2.77±2.33
G/G (n = 5)	30.80±38.70	10±13.17	2.58±0.54	2.32±3.15
*P*-value	.038*	.227	.349	.623
	rs2275913			
G/G (n = 78)	29.54±31.24	33.88±37.59	3.12±0.90	3.27±1.73
G/A (n = 61)	20.67±25.82	28.38±35.68	2.99±0.83	2.70±2.18
A/A (n = 10)	15.40±30.15	13.20±23.27	2.63±0.52	2.48±2.09
*P*-value	.012*	.115	.134	.104

CAL, clinical attachment loss; Db SNPs, single nucleotide polymorphisms; FMBS, bleeding on probing; P, probability, PD, pocket depth; rs, reference; SD, standard deviation; SNP FMPS, plaque index; p-values are unadjusted.

⁎ *P* < .05 indicates statistical significance.

#### Genetic association with periodontitis

After adjusting for confounders, GG carriers of TNF-α -238 SNP (rs361525) were significantly less likely to develop periodontitis under the dominant model (GG vs. GA + A/A: OR = 0.157, 95% CI: 0.039-0.638, *P* = .010). For OPG + 1181 (rs2073618), carriers of the CC genotype showed a significantly higher risk of periodontitis compared to GG carriers under the additive model (CC vs. GG: OR = 20.42, 95% CI: 1.95-213.9, *P* = .012). Similarly, CG carriers exhibited increased risk compared to GG carriers before adjustment (CG vs. GG: OR = 3.40, 95% CI: 1.06-10.92, *P* = .040), which further strengthened after adjustment (CG vs. GG: OR = 27.96, 95% CI: 2.51-311.38, *P* = .007). Similarly, under the recessive model, carriers of the C allele (CC + CG) showed a significantly higher risk of periodontitis compared to GG carriers after adjustment (OR = 23.20, 95% CI: 2.30-233.85, *P* = .008). For IL-1B -511 (rs16944), GG carriers were significantly less likely to develop periodontitis under both the additive (GG vs. AA: OR = 0.216, 95% CI: 0.054-0.867, *P* = .031) and dominant models (GG vs. GA + AA: OR = 0.290, 95% CI: 0.089-0.944, *P* = .040) after adjustment (Tables 3-S4). However, after applying Bonferroni correction for multiple testing across 23 SNPs (adjusted α = 0.00217), all SNPs were non-significant. Moreover, SNPs that were statistically significant in the association analyses reported in Table 3 prior to multiple-testing correction also lost significance, with all Bonferroni-adjusted *P*-values equal to 1.000 (Table S5).

**Table 3:** Association between periodontitis and SNPs using dominant, recessive, and additive models.

db SNP ID	Model (reference)	Genotypes	Odds ratio (95% CI)	*P*-value	Odds ratio (95% CI) adjusted	*P*-value adjusted
rs361525	Additive (A/A)	G/G vs A/A	-	-	-	-
	Additive (A/A)	G/A vs AA	-	-	-	-
	Dominant (G/A + A/A)	G/G vs G/A + A/A	0.412 (0.157-1.078)	.071	0.157 (0.039-0.638)	.010**
	Recessive (A/A)	G/G + G/A vs A/A	-	-	-	-
rs2073618	Additive (G/G)	C/C vs G/G	2.274 (0.728-7.10)	.157	20.416 (1.949-213.900)	.012**
	Additive (G/G)	C/G vs G/G	3.400 (1.059-10.92)	.040*	27.959 (2.510-311.376)	.007**
	Dominant (C/G + G/G)	C/C vs C/G + G/G	0.87 (0.460-1.663)	.683	1.215 (0.466-3.168)	.691
	Recessive (G/G)	C/C + C/G vs G/G	2.710 (0.904-8.123)	.075	23.195 (2.301-233.850)	.008**
rs16944	Additive (A/A)	G/G vs A/A	0.619 (0.255-1.506)	.291	0.216 (0.054-0.867)	.031**
	Additive (A/A)	G/A vs A/A	1.192 (0.536-2.649)	.667	0.619 (0.190-2.020)	.427
	Dominant (G/A + A/A)	G/G vs G/A + A/A	0.552 (0.269-1.135)	.106	0.290 (0.089-0.944)	.040**
	Recessive (A/A)	G/G + G/A vs A/A	0.931 (0.443-1.956)	.850	0.416 (0.139-1.247)	.117

CI, confidence interval; Db SNP ID, The single nucleotide polymorphisms database identifier; OR, odd ratio; P, probability; rs, reference SNP.

⁎ *P* < .05, unadjusted (crude) statistical significance.

⁎⁎ < .05, adjusted for age, sex, and smoking.

#### Genotype distribution by ethnicity and smoking status in periodontitis patients

Several SNPs showed significant differences in genotype frequencies across ethnic groups, with the SAC group exhibiting higher frequencies for most genotypes (all *P* < .05). No significant genotype differences were detected between smoking subgroups. Detailed frequencies are presented in Tables S6 & S7, respectively.

### Population stratification

The inferred cluster membership proportions indicated that individuals were evenly distributed across 2 clusters (Cluster 1: 0.507; Cluster 2: 0.493). The allele frequency divergence between clusters was moderate (F_ST = 0.1165), with mean F_ST values of 0.0957 for Cluster 1 and 0.3645 for Cluster 2. Mean expected heterozygosity within clusters was 0.3307 (Cluster 1) and 0.2424 (Cluster 2). Overall, the results suggest the presence of 2 genetically distinguishable subgroups within the cohort, though with considerable admixture (Figure S2). A sensitivity analysis excluding 1 SNP removed during QC yielded indistinguishable clustering results.

## Discussion

In this cohort, stages III and IV periodontitis predominated, reflecting a higher disease burden than reports from Egyptian, Turkish, and Norwegian populations.^24^, ^25^, ^26^ This presumably reflects the clinical hospital-based setting and underlying environmental or host factors. Grade B disease was most common, consistent with global chronic periodontitis (CP) trends, while grade C, formerly aggressive periodontitis (AgP),^27^ was less frequent, aligning with the lower proportion of Black African participants (14.6%).

Additionally, IL-17A + 197 [GA] and RANKL RL2 [GA] correlated with FMBS and FMPS, while OPG + 1181 [GC] and TNF-α −238 [GA] showed potential association with both disease susceptibility and clinical outcomes; IL-1B −511 [GA] was also linked to periodontitis risk. However, after correction for multiple testing using the Bonferroni method, none of the investigated SNP associations retained statistical significance (Table S5).

### Periodontitis clinical parameters

#### IL-17A + 197 GA (rs2275913)

No significant differences in IL-17A + 197 allele or genotype frequencies were found between cases and controls (*P =* .193), nor under any genetic model (Table S4). These results align with reports from several population studies and meta-analyses.^28^, ^29^, ^30^ Conversely, associations of the A allele or GA genotype with increased periodontitis risk were described in selected Caucasian, Asian, and mixed populations meta-analyses.^31^^,^^32^

Carriers of the GG genotype demonstrated significantly higher FMPS than AA carriers contrasting with Corrêa et al. who linked the A allele to CP and higher PPD and CAL in a Brazilian cohort, although plaque scores were not assessed.^32^ While dental plaque remains the key etiologic factor, dysbiosis rather than amount appears central to periodontitis initiation.^33^ This study therefore explored links between genetic factors and bacterial profiles, building on a pilot in the same cohort, which analysed dental plaque samples from a subset of this cohort to provide insight into the bacterial profile of periodontitis among SA.^34^ Within this infectogenomics framework, a predominance of the G allele (60% GG and 40% AG in cases vs. 60% AG and 20% GG in controls; Table S8), suggests a link between the G allele and plaque accumulation, though limited sample size may have reduced power. IL-17A cytokine plays a key role in host defence and chronic inflammation.^35^ By stimulating osteoblastic RANKL production, IL-17 shifts the OPG/RANKL balance toward bone loss.^36^ Interestingly, in this study *RANKL* and *OPG* polymorphisms were also associated with higher FMPS, highlighting their coordinated role in inflammation and bone-remodelling

#### RANKL RL2 GA (rs2277438)

The receptor activator of nuclear factor kappa-β ligand (RANKL), encoded by the TNFSF11 gene on chromosome 13, regulates alveolar bone resorption in periodontitis.^37^ In this study, no significant differences in RANKL RL2 allele or genotype frequencies were observed between cases and controls (*P* = .111), and no associations with susceptibility emerged under any genetic model, except for higher FMPS levels in AG carriers. Similar findings were reported in Iranian, Japanese, and Brazilian populations.^38^, ^39^, ^40^ Although reports on *RANKL* polymorphisms are limited, elevated RANKL levels in saliva, GCF, and gingival tissues correlate with clinical parameters, including FMPS, and bone loss.^41^, ^42^, ^43^ Increased RANKL expression in inflamed tissues suggests a role in bone destruction in AgP, though genetic links remain inconclusive.^42^

To date, no published study has examined the effect of the RL2 SNP on specific periodontal bacteria. It can be speculated that the hypothesised infectogenomics link may also apply to this variant. The RL2 SNP is located in intron 1, and such intronic variants may influence transcription or splicing, thereby affecting protein expression.^44^ In this study, AG carriers showed higher biofilm accumulation, potentially promoting dysbiosis and linking RL2 to the RANK/RANKL/OPG pathway and alveolar bone loss.^45^

### Periodontitis susceptibility and clinical parameters

#### OPG + 1181 GC (rs2073618)

Osteoprotegerin (OPG) regulates bone metabolism by competitively blocking RANK–RANKL binding on osteoclast precursors, thereby inhibiting osteoclast formation and bone resorption.^41^ Imbalances in this pathway have been demonstrated in periodontitis, with reduced OPG in GCF, saliva, and gingival tissue.^41^, ^42^, ^43^ The + 1181 SNP is located in the first exon of the TNFSF11B gene on chromosome 8, encoding the signal peptide required for OPG secretion.^46^ A G→C substitution alters the signal peptide (Lys→Asn), potentially affecting protein secretion.^47^ OPG polymorphisms have been linked to bone-related conditions such as Paget’s disease, familial expansile osteolysis, and osteoporosis, highlighting their role in bone metabolism.^48^

The role of OPG polymorphisms in osteoclastogenesis and periodontal disease is a relatively recent focus, with few studies available, mostly in limited populations,^49^ or peri-implant disease.^37^ In this study, CC genotype carriers showed a trend toward increased susceptibility to periodontitis compared to GC or GG carriers across additive and recessive models. This contrasts with findings from Korea, where the G allele predominated in periodontitis, especially in AgP cases.^50^ Furthermore, studies from Iran, Germany, and Japan reported no significant association between + 1181 SNP and periodontitis, although the CC genotype appeared more frequent in peri-implantitis than in CP or healthy groups.^39^^,^^49^^,^^51^ Consistently, studies on peri-implantitis have also shown that the CC genotype or C allele is associated with an increased risk of disease.^52^^,^^53^ Supporting this, Xu et al ^54^ found the CC genotype associated with low serum OPG levels, denoting reduced bone protection and greater susceptibility. Beyond periodontal disease, OPG + 1181, combined with RANKL RL2, correlated with low hip bone mineral density in Korean postmenopausal women.^55^

The findings of this study revealed that carriers of the CG, followed by CC genotypes, tended to exhibit higher FMPS than GG carriers (*P* = .007)**.** This pattern complements the observed genetic association of the CC genotype with higher periodontitis susceptibility, suggesting a possible dose-dependent effect of the C allele.^56^ Heterozygous CG carriers may exhibit a partial functional reduction in OPG expression, potentially contributing to increased plaque accumulation and early inflammatory activation, while homozygous CC carriers likely experience more pronounced dysregulation in bone turnover and immune response, which could predispose them to severe disease progression.

#### TNF-α -238 GA (rs361525)

The TNF-α −238 SNP showed a potential association with periodontitis, with the GG genotype observed less frequently among cases and suggesting a lower disease risk, consistent with findings from South African cohorts on SLE and obesity.^57^^,^^58^

These findings contrast with studies in multiple Asian and Caucasian populations, which reported no association with AgP or CP.^59^^,^^60^ Similarly, multi-ethnic GWAS and meta-analyses found no significant link between the −238 SNP and CP.^61^, ^62^, ^63^, ^64^ Nevertheless, Li et al ^65^ (2020) reported a positive association under recessive, over-dominant, and allele models in Asians.

GG carriers exhibited lower FMBS, a marker of inflammatory burden, than GA carriers (*P* = .043), suggesting a potential protective role and aligning with reports linking the TNF-α −308GG/−238GG haplotype to reduced bleeding in Caucasian CP patients.^66^ Conversely, carriers of the A allele or GA genotype showed a tendency toward higher FMBS, PPD, and CAL, which may reflect a genetically determined hyper-inflammatory phenotype.^67^ This suggested protective effect has been reported in psoriasis, sepsis, and OCD.^68^ Functionally, the −308G/−238G haplotype is associated with lower TNF-α expression, while A alleles increase cytokine production.^69^ As a central pro-inflammatory cytokine in periodontitis, TNF-α activity is influenced by promoter polymorphisms,^70^ though their functional impact remains debated and inconsistently reported.^66^ Additionally, -238 SNP has been proposed to be non-functional, as promoter-reporter studies showed no independent or synergistic effect on transcription.^71^ This may partially explain the observed protective trend of the wild-type G allele, predominant in healthy controls (90%), potentially reflecting reduced TNF-α transcription and cytokine output. Furthermore, given that TNF-α is located within the highly polymorphic class III region of the major histocompatibility complex (MHC) on chromosome 6, linkage disequilibrium (LD) with neighbouring HLA genes cannot be excluded particularly due to HLA’s pivotal role in immune regulation.^68^

### Periodontitis susceptibility

#### IL-1B -511 GA (rs16944)

In this study population, -511 SNP GG genotype showed a protective trend against periodontitis development, particularly after adjusting for confounders. Although widely studied, associations between −511 SNP and periodontitis remain inconsistent. Most studies across Asian, European, and multi-ethnic cohorts have reported no significant association with either CP or AgP.^72^, ^73^, ^74^, ^75^, ^76^, ^77^

Contrariwise, associations were reported when -511 formed part of haplotypes, such as IL-1B (-511 + + 3953) in Italians,^78^ IL-1A -889 + IL-1B -511 and IL-1B + 3953 + -511 in Han Chinese,^79^^,^^80^ and similar haplotypes in Macedonian, Afro-American, mulatto Brazilian, and multi-ethnic cohorts.^81^, ^82^, ^83^

Furthermore, putative population-specific protective effects of AA genotypes were also noted in South American populations^84^ and GA genotype in young Japanese females.^85^

Interestingly, IL-1B -511 protective trend extends beyond periodontitis, with GA or AA genotypes associated with reduced susceptibility to conditions such as meningococcal infection,^86^ and asthma.^87^

IL-1β is a major pro-inflammatory cytokine, consistently linked to periodontal tissue destruction.^88^ The –511 variant increased LPS-induced IL-1β secretion,^89^ illustrating pathogenic potential.

In this cohort, the observed G allele’s higher frequency among controls suggests a population-specific protective pattern, potentially reflecting unique haplotypes or gene–environment interactions influencing cytokine regulation.

Population variations in SNPs may arise from low-frequency variants (< 5%) that drive functional differences, with most diversity occurring within populations (85-90%) and less between them (10-15%),^90^ shaped by historical migration, drift, and adaptation.^91^ The uniquely admixed SA gene pool could contribute to variable SNP associations, mirroring trends in other mixed populations.^12^ In this cohort, the predominance of the SAC group may have masked interethnic differences, as admixture generates mosaic ancestry segments reflecting ancestral allele frequencies and LD patterns.^92^ Population analysis revealed 2 moderately divergent clusters with extensive admixture, reflecting the cohort’s complex ancestry and the broader SA genomic landscape.

This study had several limitations. The small sample size reduced power to detect low-frequency variants or modest effect sizes. To minimise false-positive findings in this hypothesis-driven candidate-gene study with a predefined set of SNPs, multiple testing was controlled using the Bonferroni correction, an approach commonly recommended for confirmatory analyses.^93^ While Bonferroni correction effectively controls the family-wise error rate, it is inherently conservative and may increase the risk of type II error, especially in studies with limited sample sizes.^94^ As a result, true associations of small effect may have been masked.^94^ Alternative approaches such as false discovery rate (FDR) control are often more suitable in larger or more exploratory genetic studies and could be considered in future investigations with increased sample sizes.^95^ In addition, the case–control design did not capture disease progression over time, and smoking status relied on self-report, which may introduce bias. Ancestry was self-reported and not validated using genome-wide ancestry informative markers (AIMs), which may have contributed to residual population stratification, potentially causing misclassification in this admixed population. Despite adjustment for age in all multivariable analyses, the substantial age difference between cases and controls remains a limitation. Some younger control participants may develop periodontitis later in life, which could have led to misclassification and attenuation of true genetic effects. Residual confounding by age therefore cannot be completely excluded. Potential unmeasured confounding factors, including oral hygiene behaviours, longitudinal plaque exposure, access to dental care, and comprehensive microbiome composition, were not fully captured and may have influenced observed associations. Furthermore, the lack of functional validation limits mechanistic interpretation of the observed associations. Functional studies are required to confirm the biological effects of the investigated variants, and replication in larger, ethnically diverse African cohorts is essential to establish the robustness and generalisability of these findings. Finally, the recruitment from a single province, predominantly composed of the SAC population, may restrict generalizability to other SA groups.

## Conclusion

This study provides preliminary evidence suggesting that genetic variants within OPG, TNF-α, and IL-1B may modulate susceptibility to periodontitis in a SA population. Specifically, nominal associations indicated trends toward increased disease risk for the OPG + 1181 CC genotype, while the TNF-α −238 GG and IL-1B −511 GG genotypes showed potential protective effects. However, none of these associations remained statistically significant after correction for multiple testing, and the findings should therefore be interpreted cautiously.

These findings highlight the possible population-specific patterns within an admixed African population. Larger and adequately powered studies with functional validation are required to confirm these associations and support future precision periodontal risk assessment in Africa.

## Data availability

The data that support the findings of this study are available from the corresponding author upon reasonable request.

## Authors contribution

SK: Conceptualization, Formal analysis, Investigation, Methodology, Project administration, Resources, Writing – original draft. NN: Supervision, Writing – review & editing. HH: Supervision, Writing – review & editing. YP: Supervision, Writing – review & editing. MC: Funding acquisition, Resources, Supervision, Writing – review & editing.

## Generative AI declaration of generative AI and AI-assisted technologies in the writing process

During the preparation of this work the author(s) used ChatGPT for language refinement and proofreading an entirely human-generated text, but no other use of AI was made. After using this tool, the author(s) reviewed and edited the content as needed and take(s) full responsibility for the content of the published article.

## Funding

Research reported in this study was supported by the South African Medical Research Council under a Self-Initiated Research Grant. The views and opinions expressed are those of the author(s) and do not necessarily represent the official views of the SA MRC. Additional financial support for the research, authorship, and/or publication of this article was provided by the UWC Research Chair for the SBDG project under the University Research Chairs Programme.

## Conflict of interest

The authors declare they have no conflict of interest and have no financial interest in any of the products used in this study.

## Appendix Supplementary materials

Image, application 1

## Footnotes

Supplementary material associated with this article can be found in the online version at doi:10.1016/j.identj.2026.109606.
