# Supplementary material for: Association of OPG, TNF-α, and IL-1B Gene Variants With Periodontitis in a South African Population
Source: Int Dent J. 2026 May 4;76(4):109606. doi: 10.1016/j.identj.2026.109606 (PMC13158762; doi:10.1016/j.identj.2026.109606)

Supplementary Table 1: Candidate SNPs involved in genotyping investigation for their association with periodontitis.

| **Gene** | **SNP locus** | **Alteration** | **rs number** |
| --- | --- | --- | --- |
| IL-1A | +4845 | G/T | rs17561 |
| IL-1A | - 889 | C/T | rs1800587 |
| IL-1B | +3954/3953 | C/T | rs1143634 |
| IL-1B | -31 | C/T | rs1143627 |
| IL-1B | -511 | A/G | rs16944 |
| IL-1RN | +2018 | T/C | rs419598 |
| IL-4 | -590 | C/T | rs2243250 |
| IL-4 | -33 | C/T | rs2070874 |
| IL-6 | -174 | G/C | rs1800795 |
| IL-6 | -527 | G/C | rs1800796 |
| IL-10 | -1082 | G/C | rs1800896 |
| IL-10 | -819 | C/A | rs1800871 |
| 1L-10 | -592 | C/T | rs1800872 |
| IL-17A | +197 | G/A | rs2275913 |
| TNF-α | -308 | G/A | rs1800629 |
| TNF-α | -863 | C/A | rs1800630 |
| TNF-α | -238 | G/A | rs361525 |
| IFN-𝛾 | +874 | A/T | rs2430561 |
| TLR4 | +896 | A/G | rs4986790 |
| TLR4 | +1196 | C/T | rs4986791 |
| MMP8 | -799 | C/T | rs11225395 |
| RK | RK | C/T | rs35211496 |
| RL2 | -483 | A/G | rs2277438 |
| OPG | +1181 | G/C | rs2073618 |

Supplementary table 2: Genotype and allele frequencies (counts and percentages), and p-values of HWE of the investigated SNPs in cases and controls.

| **Locus** | **dbSNP ID** | **Group** | **Genotype** | **Number (%)** | **MAF** | **Number (%)** | **HWE**  **p-value** | **Chi Squared p-value** |
| --- | --- | --- | --- | --- | --- | --- | --- | --- |
| **IL-1B**  **+3954/3953** | **rs1143634** | Cases | AA | 2 (2.6) | A | 12 (16) | 0.945 | 0.931 |
|  |  |  | AG | 20 (26.7) | G | 63 (84) |  |  |
|  |  |  | GG | 53 (70.7) |  |  |  |  |
|  |  | Controls | AA | 2 (2.7) | A | 11.3(15) | 0.721 |  |
|  |  |  | AG | 18 (24) | G | 63.7(85) |  |  |
|  |  |  | GG | 55 (73.3) |  |  |  |  |
| **IL-1A**  **-889** | **rs1800587** | Cases | AA | 12 (16) | A | 27 (36) | 0.253 | 0.301 |
|  |  |  | AG | 30 (40) | G | 48 (64) |  |  |
|  |  |  | GG | 33 (44) |  |  |  |  |
|  |  | Controls | AA | 7 (9.3) | A | 25.3(35) | 0.305 |  |
|  |  |  | AG | 38 (50.7) | G | 48.7(65) |  |  |
|  |  |  | GG | 30 (40) |  |  |  |  |
|  |  |  | AC | 31 (41.4) | C | 55.5(74) |  |  |
|  |  |  | CC | 39 (52) |  |  |  |  |

| **Locus** | **dbSNP ID** | **Group** | **Genotype** | **Number (%)** | **MAF** | **Number (%)** | **HWE**  **p-value** | **Chi Squared p-value** |
| --- | --- | --- | --- | --- | --- | --- | --- | --- |
| **IL-1A +4845** | **rs17561** | Cases | AA | 5 (6.7) | A | 25.3(35) | 0.844 | 0.935 |
|  |  |  | AC | 30 (40) | C | 48.7(65) |  |  |
|  |  |  | CC | 40 (53.3) |  |  |  |  |
|  |  | Controls | AA | 4 (5.3) | A | 19.5(26) | 0.495 |  |
|  |  |  | AC | 31 (41.4) | C | 55.5(74) |  |  |
|  |  |  | CC | 39 (52) |  |  |  |  |
| **IL-1RN +2018** | **rs419598** | Cases | CC | 0 (00) | C | 12.7(17) | 0.083 | 0.954 |
|  |  |  | CT | 25 (33.3) | T | 62.3(83) |  |  |
|  |  |  | TT | 50 (66.7) |  |  |  |  |
|  |  | Controls | CC | 0 (00) | C | 12.7(17) | 0.080 |  |
|  |  |  | CT | 26 (33.3) | T | 62.3(83) |  |  |
|  |  |  | TT | 49 (65.3) |  |  |  |  |
| **TNF-α**  **-308** | **rs1800629** | Cases | AA | 1 (1.3) | A | 8.3 (11) | 0.966 | 0.396 |
|  |  |  | AG | 15 (24) | G | 66.7(89) |  |  |
|  |  |  | GG | 59 (78.7) |  |  |  |  |
|  |  | Controls | AA | 0 (00) | A | 9.7 (13) | 0.183 |  |
|  |  |  | AG | 20 (26.7) | G | 65.3(87) |  |  |
|  |  |  | GG | 55 (73.3) |  |  |  |  |
| **TNF-α**  **-238** | **rs361525** | Cases | AA | 0 (00) | A | 7.5 (10) | 0.336 | 0.065 |
|  |  |  | AG | 15 (20) | G | 67.5(90) |  |  |
|  |  |  | GG | 60 (80) |  |  |  |  |
|  |  | Controls | AA | 0 (00) | A | 3.7 (5) | 0.672 |  |
|  |  |  | AG | 7 (9.3) | G | 71.3(95) |  |  |
|  |  |  | GG | 68 (90.7) |  |  |  |  |
| **OPG**  **+1181** | **rs2073618** | Cases | GG | 5 (6.7) | G | 21.7(29) | 0.418 | 0.098 |
|  |  |  | CG | 34 (45.3) | C | 53.3(71) |  |  |
|  |  |  | CC | 36 (48) |  |  |  |  |
|  |  | Controls | GG | 12 (16) | G | 24 (32) | 0.025* |  |
|  |  |  | CG | 24 (32) | C | 76 (68) |  |  |
|  |  |  | CC | 38 (50.7) |  |  |  |  |
| **IL-1B**  **-31** | **rs1143627** | Cases | AA | 13 (17.3) | A | 32.3(43) | 0.359 | 0.160 |
|  |  |  | AG | 41 (54.7) | G | 42.7(57) |  |  |
|  |  |  | GG | 21 (28) |  |  |  |  |
|  |  | Controls | AA | 21 (28) | A | 36.7(49) | 0.105 |  |
|  |  |  | AG | 30 (40) | G | 38.3(51) |  |  |
|  |  |  | GG | 23 (30.7) |  |  |  |  |
| **IL-1B**  **-511** | **rs16944** | Cases | AA | 19 (25.3) | A | 38.3(51) | 0.724 | 0.244 |
|  |  |  | AG | 39 (52) | G | 36.7 (49) |  |  |
|  |  |  | GG | 17 (22.7) |  |  |  |  |
|  |  | Controls | AA | 18 (24) | A | 33.7(45) | 0.156 |  |
|  |  |  | AG | 31 (41.3) | G | 41.3(55) |  |  |
|  |  |  | GG | 26 (34.7) |  |  |  |  |

| **Locus** | **dbSNP ID** | **Group** | **Genotype** | **Number (%)** | **MAF** | **Number (%)** | **HWE**  **p-value** | **Chi Squared p-value** |
| --- | --- | --- | --- | --- | --- | --- | --- | --- |
| **IL 6**  **-174** | **rs1800795** | Cases | CC | 5 (6.7) | C | 15 (20) | 0.159 | 0.933 |
|  |  |  | CG | 20 (26.7) | G | 60 (80) |  |  |
|  |  |  | GG | 49 (65.7) |  |  |  |  |
|  |  | Controls | CC | 5 (6.7) | C | 14.3(19) | 0.376 |  |
|  |  |  | CG | 20 (26.7) | G | 60.7(81) |  |  |
|  |  |  | GG | 5 (6.7) |  |  |  |  |
| **IL-10**  **-592** | **rs1800872** | Cases | TT | 7 (9.3) | G | 53.3(71) | 0.637 | 0.691 |
|  |  |  | TG | 29 (38.7) | T | 21.7(29) |  |  |
|  |  |  | GG | 39 (52) |  |  |  |  |
|  |  | Controls | TT | 7 (9.4) | G | 52.5(70) | 0.718 |  |
|  |  |  | TG | 34 (45.3) | T | 22.5(30) |  |  |
|  |  |  | GG | 34 (45.3) |  |  |  |  |
| **IL-10**  **-819** | **rs1800871** | Cases | AA | 7 (9.4) | A | 21.7(29) | 0.637 | 0.743 |
|  |  |  | AG | 29 (38.7) | G | 53.3(71) |  |  |
|  |  |  | GG | 39 (52) |  |  |  |  |
|  |  | Controls | AA | 7 (9.4) | A | 22.5(30) | 0.804 |  |
|  |  |  | AG | 33 (44) | G | 52.5(70) |  |  |
|  |  |  | GG | 34 (45.3) |  |  |  |  |
| **IL 10**  **-1082** | **rs1800896** | Cases | CC | 14 (18.7) | C | 30.7(41) | 0.572 | 0.159 |
|  |  |  | CT | 34 (45.3) | T | 44.3(59) |  |  |
|  |  |  | TT | 27 (36) |  |  |  |  |
|  |  | Controls | CC | 6 (8) | C | 24 (32) | 0.344 |  |
|  |  |  | CG | 36 (48) | T | 51 (68) |  |  |
|  |  |  | GG | 32 (42.7) |  |  |  |  |
| **MMP-8**  **-799** | **rs11225395** | Cases | AA | 7 (9.3) | A | 22.5(30) | 0.891 | 0.250 |
|  |  |  | AG | 31 (41.3) | G | 52.5(70) |  |  |
|  |  |  | GG | 37 (49.3) |  |  |  |  |
|  |  | Controls | AA | 8 (10.7) | A | 27.7(37) | 0.226 |  |
|  |  |  | AG | 40 (53.3) | G | 47.3(63) |  |  |
|  |  |  | GG | 27 (36) |  |  |  |  |
| **RANKL RL2** | **rs2277438** | Cases | GG | 49 (65.3) | A | 14.3(19) | 0.641 | 0.111 |
|  |  |  | AG | 24 (32) | G | 60.7(81) |  |  |
|  |  |  | AA | 2 (2.7) |  |  |  |  |
|  |  | Controls | GG | 59 (78.7) | A | 9.7 (13) | 0.061 |  |
|  |  |  | AG | 13 (17.3) | G | 65.3(87) |  |  |
|  |  |  | AA | 3 (4) |  |  |  |  |
| **RANK**  **RK** | **rs35211496** | Cases | TT | 0 (00) | C | 69.7(93) | 0.493 | 0.376 |
|  |  |  | TC | 11 (14.7) | T | 5.3 (7) |  |  |
|  |  |  | CC | 64 (85.3) |  |  |  |  |
|  |  | Controls | TT | 1 (1.3) | C | 70.5(94) | 0.135 |  |
|  |  |  | CT | 7 (9.3) | T | 4.5 (6) |  |  |
|  |  |  | CC | 67 (89.3) |  |  |  |  |
| **IL-4**  **-590** | **rs2243250** | Cases | CC | 28 (37.3) | C | 43.5(58) | 0.189 | 0.622 |
|  |  |  | CT | 31 (41.3) | T | 31.5(42) |  |  |
|  |  |  | TT | 16 (21.4) |  |  |  |  |
|  |  | Controls | CC | 23 (30.7) | C | 39 (52) | 0.208 |  |
|  |  |  | CT | 32 (42.7) | T | 36 (48) |  |  |
|  |  |  | TT | 20 (26.6) |  |  |  |  |

| **Locus** | **dbSNP ID** | **Group** | **Genotype** | **Number (%)** | **MAF** | **Number (%)** | **HWE**  **p-value** | **Chi Squared p-value** |
| --- | --- | --- | --- | --- | --- | --- | --- | --- |
| **IL-4**  **-33** | **rs2070874** | Cases | TT | 34 (45.3) | C | 49.5(66) | 0.494 | 0.447 |
|  |  |  | TC | 31 (41.3) | T | 25.5(34) |  |  |
|  |  |  | CC | 10 (13) |  |  |  |  |
|  |  | Controls | TT | 27 (36) | C | 44.3(59) | 0.572 |  |
|  |  |  | TC | 34 (45.3) | T | 30.7(41) |  |  |
|  |  |  | CC | 14 (18.7) |  |  |  |  |
| **IL-17A**  **+197** | **rs2275913** | Cases | AA | 3 (4) | A | 17.3(23) | 0.574 | 0.193 |
|  |  |  | AG | 28 (37.3) | G | 57.7(77) |  |  |
|  |  |  | GG | 44 (58.7) |  |  |  |  |
|  |  | Controls | AA | 7 (9.3) | A | 24 (32) | 0.804 |  |
|  |  |  | AG | 33 (44) | G | 51 (68) |  |  |
|  |  |  | GG | 34 (45.3) |  |  |  |  |
| **TLR-4**  **+896** | **rs4986790** | Cases | GG | 0 (00) | A | 71.3(95) | 0.672 | 0.979 |
|  |  |  | AG | 7 (9.3) | G | 3.7 (5) |  |  |
|  |  |  | AA | 68 (90.7) |  |  |  |  |
|  |  | Controls | GG | 0 (00) | A | 71.3(95) | 0.669 |  |
|  |  |  | AG | 7 (9.3) | G | 3.7 (5) |  |  |
|  |  |  | AA | 67 (89.3) |  |  |  |  |
| **TLR-4**  **+1196** | **rs4986791** | Cases | TT | 0 (00) | C | 71.3(95) | 0.626 | 0.807 |
|  |  |  | TC | 8 (10.7) | T | 3.7 (5) |  |  |
|  |  |  | CC | 67 (89.3) |  |  |  |  |
|  |  | Controls | TT | 0 (00) | C | 71.3(95) | 0.669 |  |
|  |  |  | TC | 7 (9.3) | T | 3.7 (5) |  |  |
|  |  |  | CC | 67 (89.3) |  |  |  |  |
| **IL-6**  **−572** | **rs1800796** | Cases | CC | 5 (6.7) | C | 13(17.3) | 0.027* | 0.561 |
|  |  |  | CG | 16 (21.3) | G | 62(82.7) |  |  |
|  |  |  | GG | 54 (72) |  |  |  |  |
|  |  | Controls | CC | 8 (10.7) | C | 18 (24) | 0.007* |  |
|  |  |  | CG | 18 (24) | G | 56 (76) |  |  |
|  |  |  | GG | 48 (64) |  |  |  |  |
| **IFN-𝛾**  **+874** | **rs2430561** | Cases | AA | 6 (8) | A | 22.5(30) | 0.680 | 0.875 |
|  |  |  | AT | 33 (44) | T | 52.5 (70 |  |  |
|  |  |  | TT | 36 (48) |  |  |  |  |
|  |  | Controls | AA | 7 (9.3) | A | 24.7(33) | 0.598 |  |
|  |  |  | AT | 35 (46.7) | T | 50.3(67) |  |  |
|  |  |  | TT | 33 (44) |  |  |  |  |
| **TNF-α**  **-863** | **rs1800630** | Cases | AA | 3 (4) | A | 16.5(22) | 0.672 | 0.756 |
|  |  |  | AC | 27 (36) | C | 58.5(78) |  |  |
|  |  |  | CC | 45 (60) |  |  |  |  |
|  |  | Controls | AA | 4 (5.3) | A | 15.7(21) | 0.575 |  |
|  |  |  | AC | 23 (30.7) | C | 59.3(79) |  |  |
|  |  |  | CC | 48 (64) |  |  |  |  |

db SNP ID: The Single Nucleotide Polymorphism Database identifier; rs: reference SNP; n: number of individuals; %: frequencies ×100; MAF: minor allele frequency ; HWE: Hardy Weinberg Equilibrium; P: probability, p-values are unadjusted; * p < 0.05 indicates statistical significance.

Supplementary Table 3: Association between SNP genotypes and clinical characteristics of periodontitis.

| **SNP**  **Genotype**  **dbSNP ID** | | **Clinical Parameters** | | | |
| --- | --- | --- | --- | --- | --- |
|  |  | **FMPS**  **(mean ± SD)** | **FMBS**  **(mean ± SD)** | **PD**  **(mean ± SD)** | **CAL**  **(mean ± SD)** |
|  |  | **rs1143634** | | | |
| GG (n = 108) | | 26.75 ± 30.54 | 30.10 ± 36.46 | 3.07 ± 0.91 | 3.04 ± 2.03 |
| GA (n = 38) | | 19.16 ± 22.58 | 29.50 ± 35.38 | 2.99 ± 0.70 | 2.85 ± 1.84 |
| AA (n = 4) | | 29.50 ± 23.60 | 33.50 ± 46.47 | 2.50 ± 0.85 | 2.83 ± 0.56 |
| p-value | | 0.335 | 0.941 | 0.438 | 0.432 |
|  | **rs1800587** | | | |  |
| GG (n = 63) | 29.56 ± 31.75 | 31.65 ± 36.51 | 3.13 ± 1.01 | 3.44 ± 1.36 |  |
| GA (n = 68) | 17.87 ± 20.38 | 25.56 ± 34.25 | 2.91 ± 0.68 | 2.50 ± 2.37 |  |
| AA (n = 19) | 34.63 ± 40.99 | 40.74 ± 40.93 | 3.16 ± 0.83 | 3.19 ± 1.67 |  |
| p-value | 0.116 | 0.149 | 0.478 | 0.282 |  |
|  | | **rs17561** | | | |
| CC (n = 79) | | 25.86 ± 29.49 | 28.90 ± 35.24 | 3.08 ± 0.99 | 3.17 ± 1.76 |
| CA (n = 61) | | 21.74 ± 25.55 | 29.25 ± 35.22 | 3.01 ± 0.67 | 2.74 ± 2.29 |
| AA (n = 9) | | 38.89 ± 46.87 | 48.78 ± 49.47 | 2.82 ± 0.76 | 3.00 ± 0.91 |
| p-value | | 0.737 | 0.649 | 0.640 | 0.521 |
|  | | **rs419598** | | | |
| TT (n = 99) | | 24.48 ± 29.68 | 32.43 ± 36.96 | 3.05 ± 0.79 | 3.07 ± 1.73 |
| TC (n = 50) | | 25.90 ± 28.78 | 25.90 ± 34.66) | 3.00 ± 0.98 | 2.82 ± 2.37 |
| p-value | | 0.851 | 0.206 | 0.437 | 0.782 |
|  | | **rs1800629** | | | |
| G/G (n = 114) | | 27.30 ± 31.58 | 31.32 ± 36.73 | 3.03 ± 0.81 | 3.02 ± 1.86 |
| G/A (n = 35) | | 16.80 ± 18.28 | 24.63 ± 34.11 | 2.98 ± 0.97 | 2.83 ± 2.26 |
| A/A (n = 1) | | 35.00 | 74.00 | 4.70 | 4.70 |
| p-value | | 0.218 | 0.433 | 0.234 | 0.296 |
|  | | **rs361525** | | | |
| G/G (n = 128) | | 24.14 ± 28.09 | 27.84 ± 35.27 | 2.99 ± 0.83 | 2.87 ± 2.03 |
| G/A (n = 22) | | 29.32 ± 35.43 | 42.86 ± 39.59 | 3.27 ± 0.98 | 3.66 ± 1.25 |
| p-value | | 0.854 | **0.043*** | 0.238 | 0.401 |
|  | | **rs2073618** | | | |
| C/C (n = 74) | | 20.57 ± 26.33 | 30.58 ± 36.88 | 3.01 ± 0.79 | 3.00 ± 1.72 |
| C/G (n = 58) | | 34.12 ± 33.58 | 33.90 ± 36.83 | 3.13 ± 0.99 | 3.03 ± 2.31 |
| G/G (n = 17) | | 12.82 ± 14.44 | 16.29 ± 29.04 | 2.79 ± 0.54 | 2.79 ± 1.75 |
| p-value | | **0.007*** | 0.281 | 0.461 | 0.231 |
|  | | **rs1143627** | | | |
| G/G (n = 44) | | 27.61 ± 32.93 | 30.68 ± 37.42 | 3.12 ± 1.02 | 3.08 ± 1.90 |
| G/A (n = 71) | | 25.96 ± 29.55 | 33.58 ± 37.90 | 3.04 ± 0.70 | 3.05 ± 1.88 |
| A/A (n = 34) | | 19.44 ± 23.25 | 22.71 ± 30.52 | 2.90 ± 0.92 | 2.71 ± 2.24 |
| p-value | | 0.564 | 0.758 | 0.316 | 0.369 |
|  | | **rs16944** | | | |
| G/G (n = 43) | | 22.35 ± 27.20 | 25.37 ± 34.16 | 2.92 ± 0.86 | 2.57 ± 2.40 |
| G/A (n = 70) | | 23.84 ± 26.98 | 31.51 ± 36.74 | 3.01 ± 0.70 | 3.12 ± 1.68 |
| A/A (n = 37) | | 29.86 ± 35.17 | 32.68 ± 37.87 | 3.20 ± 1.08 | 3.22 ± 1.85 |
| p-value | | 0.873 | 0.594 | 0.459 | 0.310 |

| **SNP**  **Genotype**  **dbSNP ID** | **Clinical Parameters** | | | |
| --- | --- | --- | --- | --- |
|  | **FMPS**  **(mean ± SD)** | **FMBS**  **(mean ± SD)** | **PD**  **(mean ± SD)** | **CAL**  **(mean ± SD)** |
|  | **rs1800795** | | | |
| G/G (n = 99) | 21.51 ± 26.81 | 29.05 ± 36.41 | 3.03 ± 0.84 | 3.01 ± 1.83 |
| G/C (n = 41) | 31.93 ± 33.64 | 31.17 ± 35.89 | 3.00 ± 0.94 | 2.75 ± 2.35 |
| C/C (n = 9) | 30.44 ± 31.54 | 31.67 ± 38.95 | 3.21 ± 0.72 | 3.73 ± 1.20 |
| p-value | 0.170 | 0.919 | 0.566 | 0.355 |
|  | **rs1800872** | | | |
| G/G (n = 73) | 25.21 ± 28.71 | 34.00 ± 39.90 | 3.10 ± 0.91 | 2.97 ± 2.12 |
| G/T (n = 63) | 23.48 ± 29.23 | 24.25 ± 32.73 | 2.89 ± 0.68 | 2.93 ± 1.72 |
| T/T (n = 14) | 29.71 ± 33.19 | 35.43 ± 40.72 | 3.31 ± 1.15 | 3.33 ± 2.13 |
| p-value | 0.533 | 0.518 | 0.297 | 0.418 |
|  | **rs1800871** | | | |
| G/G (n = 73) | 25.21 ± 28.71 | 34.00 ± 37.90 | 3.10 ± 0.91 | 2.97 ± 2.12 |
| G/A (n = 62) | 23.60 ± 29.45 | 24.64 ± 32.86 | 2.89 ± 0.69 | 2.92 ± 1.74 |
| A/A (n = 14) | 29.71 ± 33.19 | 35.43 ± 40.72 | 3.31 ± 1.15 | 3.33 ± 2.13 |
| p-value | 0.507 | 0.602 | 0.294 | 0.415 |
|  | **rs1800896** | | | |
| T/T (n = 59) | 24.73 ± 31.64 | 30.41 ± 37.84 | 3.11 ± 0.95 | 2.77 ± 2.26 |
| T/C (n = 70) | 25.11 ± 28.38 | 28.54 ± 35.20 | 2.99 ± 0.84 | 3.01 ± 1.87 |
| C/C (n = 20) | 25.40 ± 26.40 | 35.45 ± 36.40 | 3.01 ± 0.59 | 3.56 ± 1.03 |
| p-value | 0.613 | 0.573 | 0.673 | 0.389 |
|  | **rs11225395** | | | |
| G/G (n = 64) | 27.78 ± 32.08 | 32.72 ± 37.36 | 3.07 ± 0.76 | 3.16 ± 1.83 |
| G/A (n = 71) | 23.10 ± 26.66 | 28.31 ± 35.80 | 2.99 ± 0.98 | 2.82 ± 2.06 |
| A/A (n = 15) | 21.13 ± 28.69 | 26.80 ± 34.51 | 3.06 ± 0.59 | 3.01 ± 2.00 |
| p-value | 0.640 | 0.701 | 0.285 | 0.253 |
|  | **rs2277438** | | | |
| A/A (n = 108) | 20.97 ± 25.89 | 27.93 ± 35.76 | 3.04 ± 0.91 | 3.09 ± 1.75 |
| A/G (n = 37) | 35.57 ± 34.70 | 38.92 ± 38.07 | 3.06 ± 0.69 | 2.77 ± 2.33 |
| G/G (n = 5) | 30.80 ± 38.70 | 10 ± 13.17 | 2.58 ± 0.54 | 2.32 ± 3.15 |
| p-value | **0.038*** | 0.227 | 0.349 | 0.623 |
|  | **rs35211496** | | | |
| C/C (n = 131) | 25.21 ± 29.33) | 30.57 ± 36.58 | 3.04 ± 0.88 | 3.00 ± 1.94 |
| C/T (n = 18) | 23.17 ± 29.84) | 27.83 ± 34.38 | 2.96 ± 0.62 | 2.82 ± 2.16 |
| T/T (n = 1) | 16.00 | 0.00 | 3.00 | 3.20 |
| p-value | 0.841 | 0.299 | 0.993 | 0.992 |

| **SNP**  **Genotype**  **dbSNP ID** | **Clinical Parameters** | | | |
| --- | --- | --- | --- | --- |
|  | **FMPS**  **(mean ± SD)** | **FMBS**  **(mean ± SD)** | **PD**  **(mean ± SD)** | **CAL**  **(mean ± SD)** |
|  | **rs2243250** | | | |
| C/C (n = 51) | 26.73 ± 30.29 | 25.27 ± 31.33 | 3.16 ± 0.83 | 3.25 ± 1.86 |
| C/T (n = 63) | 22.35 ± 26.84 | 31.37 ± 37.45 | 2.93 ± 0.73 | 3.03 ± 1.57 |
| T/T (n = 36) | 26.78 ± 32.01 | 34.47 ± 40.39 | 3.04 ± 1.06 | 2.53 ± 2.59 |
| p-value | 0.855 | 0.859 | 0.307 | 0.336 |
|  | **rs2070874** | | | |
| C/C (n = 61) | 26.20 ± 28.39 | 26.48 ± 31.82 | 3.14 ± 0.80 | 3.29 ± 1.71 |
| C/T (n = 65) | 22.58 ± 28.68 | 32.68 ± 38.46 | 2.94 ± 0.78 | 2.98 ± 1.74 |
| T/T (n = 24) | 27.88 ± 33.24 | 31.96 ± 40.46 | 3.00 ± 1.13 | 2.22 ± 2.81 |
| p-value | 0.428 | 0.863 | 0.172 | 0.144 |
|  | **rs2275913** | | | |
| G/G (n = 78) | 29.54 ± 31.24 | 33.88 ± 37.59 | 3.12 ± 0.90 | 3.27 ± 1.73 |
| G/A (n = 61) | 20.67 ± 25.82 | 28.38 ± 35.68 | 2.99 ± 0.83 | 2.70 ± 2.18 |
| A/A (n = 10) | 15.40 ± 30.15 | 13.20 ± 23.27 | 2.63 ± 0.52 | 2.48 ± 2.09 |
| p-value | **0.012*** | 0.115 | 0.134 | 0.104 |
|  | **rs498790** | | | |
| A/A (n = 135) | 25.03 ± 29.89 | 31.37 ± 37.22 | 3.02 ± 0.85 | 2.93 ± 2.02 |
| A/G (n = 14) | 24.29 ± 23.71 | 20.29 ± 23.32 | 3.15 ± 0.96 | 3.50 ± 1.18 |
| p-value | 0.819 | 0.789 | 0.674 | 0.863 |
|  | **rs4986791** | | | |
| C/C (n = 134) | 24.82 ± 29.93 | 30.90 ± 37.04 | 3.03 ± 0.84 | 2.93 ± 1.96 |
| C/T (n = 15) | 26.20 ± 23.70 | 24.33 ± 28.18 | 3.10 ± 0.97 | 3.50 ± 1.14 |
| p-value | 0.690 | 0.972 | 0.859 | 0.812 |
|  | **rs2430561** | | | |
| T/T (n = 69) | 23.99 ± 27.08 | 33.30 ± 38.01 | 3.05 ± 0.84 | 2.96 ± 1.95 |
| T/A (n = 68) | 26.85 ± 32.47 | 28.34 ± 35.96 | 2.99 ± 0.87 | 2.97 ± 1.96 |
| A/A (n = 13) | 19.54 ± 22.20 | 21.62 ± 26.40 | 3.14 ± 0.90 | 3.19 ± 2.12 |
| p-value | 0.647 | 0.671 | 0.728 | 0.634 |
|  | **rs1800630** | | | |
| C/C (n = 93) | 25.11 ± 28.90 | 31.60 ± 38.06 | 3.03 ± 0.79 | 2.99 ± 1.87 |
| C/A (n = 50) | 26.18 ± 30.91 | 29.72 ± 34.14 | 3.04 ± 1.00 | 2.90 ± 2.24 |
| A/A (n = 7) | 13.00 ± 19.84 | 11.57 ± 11.68 | 2.99 ± 0.52 | 3.44 ± 0.45 |
| p-value | 0.315 | 0.473 | 0.902 | 0.709 |

Db SNPs: single nucleotide polymorphisms; FMPS: plaque index; FMBS: bleeding on probing; PD: pocket depth; CAL: clinical attachment loss; SD: standard deviation.

Supplementary Table 4: Association between periodontitis and SNPs using dominant, recessive, and additive models.

| **db SNP ID** | **Model (reference)** | **Genotypes** | **Odds ratio (95% CI)** | **p-value** | **Odds ratio (95% CI) adjusted** | **p-value adjusted** |
| --- | --- | --- | --- | --- | --- | --- |
| **rs1143634** | Additive (A/A) | G/G vs A/A | 0.964 (0.131 – 7.092) | 0.971 | 1.177 (0.075 – 18.393) | 0.908 |
|  | Additive (A/A) | G/A vs A/A | 1.111 (0.142 – 8.725) | 0.920 | 1.567 (0.088 – 27.853) | 0.760 |
|  | Dominant (G/A + A/A) | G/G vs G/A + A/A | 0.876 (0.429 – 1.788) | 0.716 | 0.793 (0.272 – 2.310) | 0.671 |
|  | Recessive (A/A) | G/G + G/A vs A/A | 1.000 (0.137 – 7.291) | >.999 | 1.252 (0.081 – 19.289) | 0.872 |
| **rs1800587** | Additive (A/A) | G/G vs A/A | 0.642 (0.223 – 1.843) | 0.410 | 0.851 (0.190 – 3.819) | 0.833 |
|  | Additive (A/A) | G/A vs A/A | 0.461 (0.161 – 1.313) | 0.147 | 0.749 (0.171 – 3.275) | 0.701 |
|  | Dominant (G/A + A/A) | G/G vs G/A + A/A | 1.179 (0.616 – 2.255) | 0.620 | 1.064 (0.400 – 2.829) | 0.901 |
|  | Recessive (A/A) | G/G + G/A vs A/A | 0.540 (0.200 – 1.459) | 0.225 | 0.795 (0.197 – 3.208) | 0.747 |
| **rs17561** | Additive (A/A) | C/C vs A/A | 0.821 (0.205 – 3.284) | 0.780 | 1.240 (0.149 – 10.348) | 0.843 |
|  | Additive (A/A) | C/A vs A/A | 0.774 (0.190 – 3.163) | 0.722 | 1.883 (0.217 – 16.361) | 0.566 |
|  | Dominant (C/A + A/A) | C/C vs C/A + A/A | 1.026 (0.539 – 1.952) | 0.939 | 0.713 (0.272 – 1.872) | 0.493 |
|  | Recessive (A/A) | C/C + C/A vs A/A | 0.800 (0.206 – 3.104) | 0.747 | 1.477 (0.186 – 11.726) | 0.712 |
| **rs419598** | Additive (C/C) | T/T vs C/C | - | - | - | - |
|  | Additive (C/C) | T/C vs C/C | - | - | - | - |
|  | Dominant (C/T + C/C) | T/T vs C/T + C/C | 1.020 (0.517 – 2.014) | 0.954 | 2.139 (0.703 – 6.508) | 0.180 |
|  | Recessive (C/C) | T/T + C/T vs C/C | - | - | - | - |
| **rs1800629** | Additive (A/A) | G/G vs A/A | - | - | - | - |
|  | Additive (A/A) | G/A vs A/A | - | - | - | - |
|  | Dominant (G/A + A/A) | G/G vs G/A + A/A | 1.341 (0.631 – 2.847) | 0.445 | 0.999 (0.336 – 2.973) | 0.999 |
|  | Recessive (A/A) | G/G + G/A vs A/A | - | - | - | - |

Supplementary Table 4 (continued): Association between periodontitis and SNPs using dominant, recessive, and additive models. models.

| **db SNP ID** | **Model (reference)** | **Genotypes** | **Odds ratio (95% CI)** | **p-value** | **Odds ratio (95% CI) adjusted** | **p-value adjusted** |
| --- | --- | --- | --- | --- | --- | --- |
| **rs361525** | Additive (A/A) | G/G vs A/A | - | - | - | - |
|  | Additive (A/A) | G/A vs AA | - | - | - | - |
|  | Dominant (G/A + A/A) | G/G vs G/A + A/A | 0.412 (0.157 – 1.078) | 0.071 | 0.157 (0.039 – 0.638) | **0.010**** |
|  | Recessive (A/A) | G/G + G/A vs A/A | - | - | - | - |
| **rs2073618** | Additive (G/G) | C/C vs G/G | 2.274 (0.728 – 7.10) | 0.157 | 20.416 (1.949 – 213.900) | **0.012**** |
|  | Additive (G/G) | C/G vs G/G | 3.400 (1.059 – 10.92) | **0.040*** | 27.959 (2.510 – 311.376) | **0.007**** |
|  | Dominant (C/G + G/G) | C/C vs C/G + G/G | 0.87 (0.460 – 1.663) | 0.683 | 1.215 (0.466 – 3.168) | 0.691 |
|  | Recessive (G/G) | C/C + C/G vs G/G | 2.710 (0.904 – 8.123) | 0.075 | 23.195 (2.301 – 233.850) | **0.008**** |
| **rs1143627** | Additive (A/A) | G/G vs A/A | 1.475 (0.594 – 3.665) | 0.403 | 3.722 (0.893 – 15.512) | 0.071 |
|  | Additive (A/A) | G/A vs A/A | 2.208 (0.956 – 5.097) | 0.064 | 2.913 (0.755 – 11.238) | 0.121 |
|  | Dominant (G/A + A/A) | G/G vs G/A + A/A | 0.862 (0.426 – 1.744) | 0.680 | 1.772 (0.635 – 4950) | 0.275 |
|  | Recessive (A/A) | G/G + G/A vs A/A | 1.890 (0.864 – 4.134) | 0.111 | 3.224 (0.906 – 11.478) | 0.071 |
| **rs16944** | Additive (A/A) | G/G vs A/A | 0.619 (0.255 – 1.506) | 0.291 | 0.216 (0.054 – 0.867) | **0.031**** |
|  | Additive (A/A) | G/A vs A/A | 1.192 (0.536 – 2.649) | 0.667 | 0.619 (0.190 – 2.020) | 0.427 |
|  | Dominant (G/A + A/A) | G/G vs G/A + A/A | 0.552 (0.269 – 1.135) | 0.106 | 0.290 (0.089 – 0.944) | **0.040**** |
|  | Recessive (A/A) | G/G + G/A vs A/A | 0.931 (0.443 – 1.956) | 0.850 | 0.416 (0.139 – 1.247) | 0.117 |
| **rs1800795** | Additive (C/C) | G/G vs C/C | 0.784 (0.199 – 3.093) | 0.728 | 2.487 (0.248 – 24.975) | 0.439 |
|  | Additive (C/C) | G/C vs C/C | 0.762 (0.179 – 3.249) | 0.713 | 1.682 (0.154 – 18.374) | 0.670 |
|  | Dominant (G/C + C/C) | G/G vs G/C + C/C | 0.980 (0.496 – 1.935) | 0.954 | 1.599 (0.550 – 4.649) | 0.389 |
|  | Recessive (C/C) | G/G + G/C vs C/C | 0.777 (0.200 – 3.017) | 0.716 | 2.179 (0.227 – 20.899) | 0.500 |

Supplementary Table 4 (continued): Association between periodontitis and SNPs using dominant, recessive, and additive models.

| **db SNP ID** | **Model (reference)** | **Genotypes** | **Odds ratio (95% CI)** | **p-value** | **Odds ratio (95% CI) adjusted** | **p-value adjusted** |
| --- | --- | --- | --- | --- | --- | --- |
| **rs1800872** | Additive (T/T) | G/G vs. T/T | 1.147 (0.365 – 3.601) | 0.814 | 2.563 (0.318 – 20.691) | 0.377 |
|  | Additive (T/T) | G/T vs. T/T | 0.853 (0.268 – 2.718) | 0.788 | 2.004 (0.239 – 16.778) | 0.522 |
|  | Dominant (G/T + T/T) | G/G vs. G/T + T/T | 1.306 (0.688 – 2.482) | 0.414 | 1.409 (0.523 – 3.793) | 0.498 |
|  | Recessive (T/T) | G/G + G/T vs. T/T | 1.000 (0.333 – 3.005) | >0.999 | 2.310 (0.279 – 17.950) | 0.424 |
| **rs1800871** | Additive (A/A) | G/G vs. A/A | 1.147 (0.365 – 3.601) | 0.814 | 2.552 (0.319 – 20.454) | 0.378 |
|  | Additive (A/A) | G/A vs. A/A | 0.879 (0.275 – 2.804) | 0.827 | 2.023 (0.243 – 16.830) | 0.514 |
|  | Dominant (G/A + A/A) | G/G vs. G/A + A/A | 1.275 (0.670 – 2.426) | 0.460 | 1.393 (0.517 – 3.749) | 0.512 |
|  | Recessive (A/A) | G/G + G/A vs. A/A | 1.015 (0.338 – 3.051) | 0.979 | 2.314 (0.300 – 17.833) | 0.421 |
| **rs1800896** | Additive (C/C) | T/T vs. C/C | 0.362 (0.122 – 1.070) | 0.066 | 0.535 (0.125 – 2.294) | 0400 |
|  | Additive (C/C) | T/C vs. C/C | 0.405 (0.140 – 1.174) | 0.096 | 0.449 (0.105 – 1.921) | 0.280 |
|  | Dominant (T/C + C/C) | T/T vs. T/C + C/C | 0.738 (0.382 – 1.426) | 0.367 | 0.960 (0.357 – 2.579) | 0.935 |
|  | Recessive (C/C) | T/T + T/C vs. C/C | 0.384 (0.139 – 1.063) | 0.065 | 0.489 (0.126 – 1.895) | 0.301 |
| **rs11225395** | Additive (A/A) | G/G vs. A/A | 1.566 (0.506 – 4.84) | 0.436 | 2.795 (0.336 – 23.264) | 0.342 |
|  | Additive (A/A) | G/A vs. A/A | 0.886 (0.290 – 2.708) | 0.831 | 2.354 (0.280 – 19.807) | 0.431 |
|  | Dominant (G/A + A/A) | G/G vs. G/A + A/A | 1.731 (0.900 – 3.328) | 0.100 | 1.318 (0.496 – 3.505) | 0.580 |
|  | Recessive (A/A) | G/G + G/A vs. A/A | 1.160 (0.398 – 3.378) | 0.786 | 3.224 (0.906 – 11.478) | 0.071 |
| **rs2277438** | Additive (G/G) | A/A vs. G/G | 1.246 (0.200 – 7.757) | 0.814 | 3.979 (0.023 – 682.884) | 0.599 |
|  | Additive (G/G) | A/G vs. G/G | 2.769 (0.409 – 18.743) | 0.296 | 6.451 (0.035 – 1174.847) | 0.483 |
|  | Dominant (A/G + G/G) | A/A vs. A/G + G/G | 0.511 (0.247 – 1.059) | 0.071 | 0.679 (0.225 – 2.047) | 0.491 |
|  | Recessive (G/G) | A/A + A/G vs. G/G | 1.521 (0.247 – 9.373) | 0.651 | 4.548 (0.025 – 824.346) | 0.568 |

Supplementary Table 4 (continued): Association between periodontitis and SNPs using dominant, recessive, and additive models.

| **db SNP ID** | **Model (reference)** | **Genotypes** | **Odds ratio (95% CI)** | **p-value** | **Odds ratio (95% CI) adjusted** | **p-value adjusted** |
| --- | --- | --- | --- | --- | --- | --- |
| **rs35211496** | Additive (T/T) | C/C vs. T/T | - | - | - | - |
|  | Additive (T/T) | C/T vs. T/T | - | - | - | - |
|  | Dominant (C/T + T/T) | C/C vs. C/T + T/T | 0.695 (0.263 – 1.838) | 0.463 | 1.799 (0.317 – 10.212) | 0.508 |
|  | Recessive (T/T) | C/C + C/T vs. T/T | - | - | - | - |
| **rs2243250** | Additive (T/T) | C/C vs. T/T | 1.522 (0.645 – 3.589) | 0.338 | 0.644 (0.185 – 2.242) | 0.490 |
|  | Additive (T/T) | C/T vs. T/T | 1.211 (0.532 – 2.756) | 0.648 | 0.494 (0.145 – 1.691) | 0.261 |
|  | Dominant (C/T + T/T) | C/C vs. C/T + T/T | 1.347 (0.684 – 2.654) | 0.389 | 0.970 (0.348 – 2.702) | 0.953 |
|  | Recessive (T/T) | C/C + C/T vs. T/T | 1.341 (0.631 – 2.847) | 0.445 | 0.560 (0.118 – 1.671) | 0.99 |
| **rs2070874** | Additive (T/T) | C/C vs. T/T | 1.763 (0.678 – 4.586) | 0.245 | 1.511 (0.371 – 6.151) | 0.564 |
|  | Additive (T/T) | C/T vs. T/T | 1.276 (0.496 – 3.288) | 0.613 | 1.249 (0.307 – 5.052) | 0.755 |
|  | Dominant (C/T + T/T) | C/C vs. C/T + T/T | 1.474 (0.766 – 2.838) | 0.245 | 1.290 (0.484 – 3.441) | 0.611 |
|  | Recessive (T/T) | C/C + C/T vs. T/T | 1.492 (0.617 – 3.609) | 0.375 | 1.371 (0.375 – 5.016) | 0.633 |
| **rs2275913** | Additive (A/A) | G/G vs. A/A | 3.020 (0.727 – 12.550) | 0.128 | 9.389 (0.799 – 110.380) | 0.075 |
|  | Additive (A/A) | G/A vs. A/A | 1.980 (0.468 – 8.383) | 0.354 | 4.968 (0.414 – 59.588) | 0.206 |
|  | Dominant (G/A + A/A) | G/G vs. G/A + A/A | 1.670 (0.873 – 3.193) | 0.121 | 2.268 (0.825 – 6.237) | 0.113 |
|  | Recessive (A/A) | G/G + G/A vs. A/A | 2.507 (0.623 – 10.096) | 0.196 | 7.224 (0.636 – 82.029) | 0.111 |
| **rs4986790** | Additive (G/G) | A/A vs. G/G |  |  |  |  |
|  | Additive (G/G) | A/G vs. G/G | - | - | - | - |
|  | Dominant (A/G) | A/A vs. A/G + G/G | 1.015 (0.338 – 3.051) | 0.979 | 2.582 (0.316 – 21.103) | 0.376 |
|  | Recessive (G/G) | A/A + A/G vs. G/G | - | - | - | - |

Supplementary Table 4 (continued): Association between periodontitis and SNPs using dominant, recessive, and additive models.

| **db SNP ID** | **Model (reference)** | **Genotypes** | **Odds ratio (95% CI)** | **p-value** | **Odds ratio (95% CI) adjusted** | **p-value adjusted** |
| --- | --- | --- | --- | --- | --- | --- |
| **rs4986791** | Additive (T/T) | C/C vs. T/T |  |  |  |  |
|  | Additive (T/T) | C/T vs. T/T | - | - | - | - |
|  | Dominant (C/T + T/T) | C/C vs. C/T + T/T | 0.875 (0.300 – 2.549) | 0.807 | 3.762 (0.476 – 29.747) | 0.209 |
|  | Recessive (T/T) | C/C + C/T vs. T/T | - | - | - | - |
| **rs2430561** | Additive (C/C) | G/G vs. C/C | 1.800 (0.551 – 5.876) | 0.330 | 2.463 (0.291 – 20.818) | 0.408 |
|  | Additive (C/C) | G/C vs. C/C | 1.422 (0.386 – 5.243) | 0.597 | 1.291 (0.128 – 13.007) | 0.829 |
|  | Dominant (G/C + C/C) | G/G vs. G/C + C/C | 1.393 (0.696 – 2.789) | 0.349 | 2.013 (0.682 – 5.945) | 0.205 |
|  | Recessive C/C) | G/G + G/C vs. C/C | 1.697 (0.528 – 5.450) | 0.374 | 2.142 (0.262 – 17.521) | 0.447 |
| **rs1800630** | Additive (A/A) | T/T vs. A/A | 1.273 (0.388 – 4.177) | 0.691 | 2.109 (0.230 – 19.303) | 0.509 |
|  | Additive (A/A) | T/A vs. A/A | 1.100 (0.335 – 3.614) | 0.875 | 2.038 (0.221 – 18.5769) | 0.530 |
|  | Dominant (T/A + A/A) | T/T vs. T/A + A/A | 1.175 (0.618 – 2.234) | 0.623 | 1.118 (0.425 – 2.940) | 0.821 |
|  | Recessive (A/A) | T/T + T/A vs. A/A | 1.184 (0.378 – 3.704) | 0.772 | 2.074 (0.239 – 17.998) | 0.508 |

Db SNP ID: The single nucleotide polymorphisms database identifier; rs: reference SNP; OR: odd ratio; CI: confidence interval; P: probability;*: p < 0.05, unadjusted (crude) statistical significance;**: p < 0.05, adjusted for age, sex, and smoking.

Supplementary Table 5: Results of Bonferroni correction for multiple testing across 23 SNPs (adjusted α = 0.00217). None of the associations remained statistically significant after correction.

| SNP_ID | Model_used | | Raw_p_value | | N_tests_m | | Alpha_family | | Alpha_per_test | | | P_bonf | Significant | |
| --- | --- | --- | --- | --- | --- | --- | --- | --- | --- | --- | --- | --- | --- | --- |
| rs1143634 | | Additive (adjusted) | | *0.908* | | 69 | | 0.05 | | *0.00072* | 1 | | | FALSE |
| rs1143634 | | Dominant | | *0.671* | | 69 | | 0.05 | |  | 1 | | | FALSE |
| rs1143634 | | Recessive | | *0.872* | | 69 | | 0.05 | | *0.00072* | 1 | | | FALSE |
| rs1800587 | | Additive (adjusted) | | *0.833* | | 69 | | 0.05 | |  | 1 | | | FALSE |
| rs1800587 | | Dominant | | *0.901* | | 69 | | 0.05 | | *0.00072* | 1 | | | FALSE |
| rs1800587 | | Recessive | | *0.747* | | 69 | | 0.05 | |  | 1 | | | FALSE |
| rs17561 | | Additive (adjusted) | | *0.843* | | 69 | | 0.05 | | *0.00072* | 1 | | | FALSE |
| rs17561 | | Dominant | | *0.493* | | 69 | | 0.05 | |  | 1 | | | FALSE |
| rs17561 | | Recessive | | *0.712* | | 69 | | 0.05 | | *0.00072* | 1 | | | FALSE |
| rs419598 | | Additive (adjusted) | | | | 69 | | 0.05 | |  | 1 | | | FALSE |
| rs419598 | | Dominant | | *0.18* | | 69 | | 0.05 | | *0.00072* | 1 | | | FALSE |
| rs419598 | | Recessive | |  | | 69 | | 0.05 | |  | 1 | | | FALSE |
| rs1800629 | | Additive (adjusted) | | | | 69 | | 0.05 | | *0.00072* | 1 | | | FALSE |
| rs1800629 | | Dominant | | 0.999 | | 69 | | 0.05 | |  | 1 | | | FALSE |
| rs1800629 | | Recessive | |  | | 69 | | 0.05 | | *0.00072* | 1 | | | FALSE |
| rs361525 | | Additive (adjusted) | | | | 69 | | 0.05 | |  | 1 | | | FALSE |
| rs361525 | | Dominant | | *0.01* | | 69 | | 0.05 | | *0.00072* | 1 | | | FALSE |
| rs361525 | | Recessive | |  | | 69 | | 0.05 | |  | 1 | | | FALSE |
| rs2073618 | | Additive (adjusted) | | *0.012* | | 69 | | 0.05 | | *0.00072* | 1 | | | FALSE |
| rs2073618 | | Dominant | | *0.691* | | 69 | | 0.05 | |  | 1 | | | FALSE |
| rs2073618 | | Recessive | | *0.008* | | 69 | | 0.05 | | *0.00072* | 1 | | | FALSE |
| rs1143627 | | Additive (adjusted) | | *0.071* | | 69 | | 0.05 | |  | 1 | | | FALSE |
| rs1143627 | | Dominant | | *0.275* | | 69 | | 0.05 | | *0.00072* | 1 | | | FALSE |
| rs1143627 | | Recessive | | *0.071* | | 69 | | 0.05 | |  | 1 | | | FALSE |
| rs16944 | | Additive (adjusted) | | *0.031* | | 69 | | 0.05 | | *0.00072* | 1 | | | FALSE |
| rs16944 | | Dominant | | *47* | | 69 | | 0.05 | |  | 1 | | | FALSE |
| rs16944 | | Recessive | | *0.117* | | 69 | | 0.05 | | *0.00072* | 1 | | | FALSE |
| rs1800795 | | Additive (adjusted) | | *0.439* | | 69 | | 0.05 | |  | 1 | | | FALSE |
| rs1800795 | | Dominant | | *0.389* | | 69 | | 0.05 | | *0.00072* | 1 | | | FALSE |
| rs1800795 | | Recessive | | *0.117* | | 69 | | 0.05 | |  | 1 | | | FALSE |
| rs1800872 | | Additive (adjusted) | | *0.5* | | 69 | | 0.05 | | *0.00072* | 1 | | | FALSE |
| rs1800872 | | Dominant | | *0.337* | | 69 | | 0.05 | |  | 1 | | | FALSE |
| rs1800872 | | Recessive | |  | | 69 | | 0.05 | | *0.00072* | 1 | | | FALSE |
| rs1800871 | | Additive (adjusted) | | | | 69 | | 0.05 | |  | 1 | | | FALSE |
| rs1800871 | | Dominant | |  | | 69 | | 0.05 | | *0.00072* | 1 | | | FALSE |
| rs1800871 | | Recessive | |  | | 69 | | 0.05 | |  | 1 | | | FALSE |
| rs1800896 | | Additive (adjusted) | | | | 69 | | 0.05 | | *0.00072* | 1 | | | FALSE |
| rs1800896 | | Dominant | |  | | 69 | | 0.05 | |  | 1 | | | FALSE |
| rs1800896 | | Recessive | |  | | 69 | | 0.05 | | *0.00072* | 1 | | | FALSE |
| rs11225395 | | Additive (adjusted) | | | | 69 | | 0.05 | |  | 1 | | | FALSE |
| rs11225395 | | Dominant | |  | | 69 | | 0.05 | | *0.00072* | 1 | | | FALSE |
| rs11225395 | | Recessive | |  | | 69 | | 0.05 | |  | 1 | | | FALSE |
| rs2277438 | | Additive (adjusted) | | | | 69 | | 0.05 | | *0.00072* | 1 | | | FALSE |
| rs2277438 | | Dominant | |  | | 69 | | 0.05 | |  | 1 | | | FALSE |
| rs2277438 | | Recessive | |  | | 69 | | 0.05 | | *0.00072* | 1 | | | FALSE |
| rs35211496 | | Additive (adjusted) | | | | 69 | | 0.05 | |  | 1 | | | FALSE |
| rs35211496 | | Dominant | |  | | 69 | | 0.05 | | *0.00072* | 1 | | | FALSE |
| rs35211496 | | Recessive | |  | | 69 | | 0.05 | |  | 1 | | | FALSE |
| rs2243250 | | Additive (adjusted) | | | | 69 | | 0.05 | | *0.00072* | 1 | | | FALSE |
| rs2243250 | | Dominant | |  | | 69 | | 0.05 | |  | 1 | | | FALSE |
| rs2243250 | | Recessive | |  | | 69 | | 0.05 | | *0.00072* | 1 | | | FALSE |
| rs2070874 | | Additive (adjusted) | | | | 69 | | 0.05 | |  | 1 | | | FALSE |
| rs2070874 | | Dominant | |  | | 69 | | 0.05 | | *0.00072* | 1 | | | FALSE |
| rs2070874 | | Recessive | |  | | 69 | | 0.05 | |  | 1 | | | FALSE |
| rs2275913 | | Additive (adjusted) | | | | 69 | | 0.05 | | *0.00072* | 1 | | | FALSE |
| rs2275913 | | Dominant | |  | | 69 | | 0.05 | |  | 1 | | | FALSE |
| rs2275913 | | Recessive | |  | | 69 | | 0.05 | | *0.00072* | 1 | | | FALSE |
| rs4986790 | | Additive (adjusted) | | | | 69 | | 0.05 | |  | 1 | | | FALSE |
| rs4986790 | | Dominant | |  | | 69 | | 0.05 | | *0.00072* | 1 | | | FALSE |
| rs4986790 | | Recessive | |  | | 69 | | 0.05 | |  | 1 | | | FALSE |
| rs4986791 | | Additive (adjusted) | | | | 69 | | 0.05 | | *0.00072* | 1 | | | FALSE |
| rs4986791 | | Dominant | |  | | 69 | | 0.05 | |  | 1 | | | FALSE |
| rs4986791 | | Recessive | |  | | 69 | | 0.05 | | *0.00072* | 1 | | | FALSE |
| rs2430561 | | Additive (adjusted) | | | | 69 | | 0.05 | |  | 1 | | | FALSE |
| rs2430561 | | Dominant | |  | | 69 | | 0.05 | | *0.00072* | 1 | | | FALSE |
| rs2430561 | | Recessive | |  | | 69 | | 0.05 | |  | 1 | | | FALSE |
| rs1800630 | | Additive (adjusted) | | | | 69 | | 0.05 | | *0.00072* | 1 | | | FALSE |
| rs1800630 | | Dominant | |  | | 69 | | 0.05 | |  | 1 | | | FALSE |
| rs1800630 | | Recessive | |  | | 69 | | 0.05 | | *0.00072* | 1 | | | FALSE |

SNP_ID: single nucleotide polymorphism identifier; Model used: genetic model applied in association testing; Raw p value: unadjusted p-value from association analysis; N tests (m): total number of tests performed; Alpha family: family-wise significance level (α = 0.05); Alpha per test: Bonferroni-adjusted significance threshold (α/m); P_bonf: Bonferroni-adjusted p-value; Significant: statistical significance after Bonferroni correction (p < 0.00217).

Supplementary Table 6: The association between investigated SNPs and periodontitis across different ethnicities.

| **Ethnicity**  **SNPs**  **Genotypes** | **African**  **N (%)** | **Caucasian**  **N (%)** | **Asian/Indian**  **N (%)** | **SAC**  **N (%)** |
| --- | --- | --- | --- | --- |
| **rs1143634** | | | | |
| GG (n = 106) | 24 (22.6) | 17 (16.1) | 9 (8.5) | 56 (52.8) |
| GA (n = 38) | 5 (13.2) | 13 (34.2) | 9 (23.7) | 11 (28.9) |
| AA (n = 4) | 0 (0) | 3 (75.0) | 0 (0) | 1 (25.0) |
| p-value | 0.002* | | | |

| **Ethnicity**  **SNPs**  **Genotypes** | **African**  **N (%)** | **Caucasian**  **N (%)** | **Asian/Indian**  **N (%)** | **SAC**  **N (%)** |
| --- | --- | --- | --- | --- |
| **rs1800587** | | | | |
| GG (n = 62) | 11 (17.7) | 10 (16.1) | 4 (6.5) | 37 (59.7) |
| GA (n = 67) | 14 (20.9) | 16 (23.9) | 14 (20.9) | 23 (34.3) |
| AA (n = 19) | 4 (21.1) | 7 (36.8) | 0 (0) | 8 (42.1) |
| p-value | 0.014* | | | |
| **rs17561** | | | | |
| CC (n = 78) | 17 (21.8) | 10 (12.8) | 5 (6.4) | 46 (59) |
| CA (n = 60) | 12 (20) | 16 (26.6) | 13 (21.7) | 19 (31.7) |
| AA (n = 9) | 0 (0) | 6 (66.7) | 0 (0) | 3 (33.3) |
| p-value | <0.001* | | | |
| **rs419598** | | | | |
| TT (n = 97) | 24 (24.7) | 16 (16.5) | 11 (11.4) | 46 (47.4) |
| TC (n = 50) | 5 (10) | 16 (32) | 7 (14) | 22 (44) |
| p-value | 0.056 | | | |
| **rs1800629** | | | | |
| G/G (n = 113) | 18 (16) | 21 (18.6) | 16 (14.1) | 58 (51.3) |
| G/A (n = 34) | 11 (32.3) | 12 (35.3) | 2 (5.9) | 9 (26.5) |
| A/A (n = 1) | 0 (0) | 0 (0) | 0 (0) | 1 (100) |
| p-value | 0.042* | | | |
| **rs361525** | | | | |
| G/G (n = 127) | 24 (18.9) | 29 (22.8) | 16 (12.6) | 58 (45.7) |
| G/A (n = 21) | 5 (23.8) | 4 (19.1) | 2 (9.5) | 10 (47.6) |
| p-value | 0.920 | | | |
| **rs2073618** | | | | |
| C/C (n = 73) | 25 (34.2) | 13 (17.8) | 7 (9.6) | 28 (38.4) |
| C/G (n = 58) | 4 (6.9) | 15 (25.9) | 7 (12) | 32 (55.2) |
| G/G (n = 16) | 0 (0) | 4 (25) | 4 (25) | 8 (50) |
| p-value | 0.002* | | | |
| **rs1143627** | | | | |
| G/G (n = 43) | 14 (32.6) | 2 (4.6) | 5 (11.6) | 22 (51.2) |
| G/A (n = 71) | 11 (15.5) | 18 (25.4) | 6 (8.5) | 36 (50.6) |
| A/A (n = 33) | 4 (12.1) | 12 (36.4) | 7 (21.2) | 10 (30.3) |
| p-value | 0.003* | | | |
| **rs16944** | | | | |
| G/G (n = 42) | 7 (16.7) | 13 (31) | 7 (16.7) | 15 (35.6) |
| G/A (n = 70) | 11 (15.7) | 18 (25.7) | 7 (10) | 34 (48.6) |
| A/A (n = 36) | 11 (30.6) | 2 (5.6) | 4 (11.1) | 19 (52.7) |
| p-value | 0.070 | | | |

| **Ethnicity**  **SNPs**  **Genotypes** | **African**  **N (%)** | **Caucasian**  **N (%)** | **Asian/Indian**  **N (%)** | **SAC**  **N (%)** |
| --- | --- | --- | --- | --- |
| **rs1800795** | | | | |
| G/G (n = 97) | 29 (29.9) | 12 (12.4) | 9 (9.3) | 47 (48.4) |
| G/C (n = 41) | 0 (0) | 16 (39) | 8 (19.5) | 17 (41.5) |
| C/C (n = 9) | 0 (0) | 5 (55.6) | 1 (11.1) | 3 (33.3) |
| p-value | <0.001* | | | |
| **rs1800872** | | | | |
| G/G (n = 73) | 15 (20.5) | 20 (27.4) | 8 (11) | 30 (41.1) |
| G/T (n = 61) | 12 (19.7) | 13 (21.3) | 8 (13.1) | 28 (45.9) |
| T/T (n = 14) | 2 (14.3) | 0 (0) | 2 (14.3) | 10 (71.4) |
| p-value | 0.342 | | | |
| **rs1800871** | | | | |
| G/G (n = 73) | 15 (20.5) | 20 (27.4) | 8 (11) | 30 (41.1) |
| G/A (n = 60) | 12 (20) | 12 (20) | 8 (13.3) | 28 (46.7) |
| A/A (n = 14) | 2 (14.3) | 0 (0) | 2 (14.3) | 10 (71.4) |
| p-value | 0.323 | | | |
| **rs1800896** | | | | |
| T/T (n = 59) | 14 (23.7) | 10 (17) | 10 (17) | 25 (42.4) |
| T/C (n = 68) | 12 (17.6) | 17 (25) | 6 (8.8) | 33 (48.6) |
| C/C (n = 20) | 2 (10) | 6 (30) | 2 (10) | 10 (50) |
| p-value | 0.514 | | | |
| **rs11225395** | | | | |
| G/G (n = 63) | 14 (22.2) | 10 (15.9) | 7 (11.1) | 32 (50.8) |
| G/A (n = 70) | 14 (20) | 17 (24.3) | 8 (11.4) | 31 (44.3) |
| A/A (n = 15) | 1 (6.7) | 6 (40) | 3 (20) | 5 (33.3) |
| p-value | 0.356 | | | |
| **rs2277438** | | | | |
| A/A (n = 106) | 23 (21.7) | 19 (17.9) | 14 (13.2) | 50 (47.2) |
| A/G (n = 37) | 6 (16.2) | 12 (32.4) | 3 (8.1) | 16 (43.2) |
| G/G (n = 5) | 0 (0) | 2 (40) | 1 (20) | 2 (40) |
| p-value | 0.454 | | | |
| **rs35211496** | | | | |
| C/C (n = 129) | 29 (22.1) | 21 (16) | 17 (13) | 62 (47.3) |
| C/T (n = 18) | 0 (0) | 11 (61.1) | 1 (5.6) | 6 (33.3) |
| T/T (n = 1) | 0 (0) | 1 (100) | 0 (0) | 0 (0) |
| p-value | <0.001* | | | |

| **Ethnicity**  **SNPs**  **Genotypes** | **African**  **N (%)** | **Caucasian**  **N (%)** | **Asian/Indian**  **N (%)** | **SAC**  **N (%)** |
| --- | --- | --- | --- | --- |
| **rs2243250** | | | | |
| C/C (n = 49) | 2 (4.1) | 19 (38.8) | 8 (16.3) | 20 (40.8) |
| C/T (n = 63) | 7 (11.1) | 13 (20.6) | 10 (15.9) | 33 (52.4) |
| T/T (n = 36) | 20 (55.6) | 1 (2.7) | 0 (0) | 15 (41.7) |
| p-value | <0.001* | | | |
| **rs2070874** | | | | |
| C/C (n = 59) | 3 (5.1) | 20 (33.9) | 8 (13.5) | 28 (47.5) |
| C/T (n = 65) | 16 (24.6) | 12 (18.5) | 10 (15.4) | 27 (41.5) |
| T/T (n = 24) | 10 (41.7) | 1 (4.2) | 0 (0) | 13 (54.1) |
| p-value | <0.001* | | | |
| **rs2275913** | | | | |
| G/G (n = 77) | 25 (32.5) | 12 (15.5) | 5 (6.5) | 35 (45.5) |
| G/A (n = 60) | 4 (6.6) | 16 (26.7) | 13 (21.7) | 27 (45) |
| A/A (n = 10) | 0 (0) | 4 (40) | 0 (0) | 6 (60) |
| p-value | <0.001* | | | |
| **rs4986790** | | | | |
| A/A (n = 133) | 28 (21.1) | 30 (22.5) | 16 (12) | 59 (44.4) |
| A/G (n = 14) | 1 (7.1) | 2 (14.3) | 2 (14.3) | 9 (64.3) |
| p-value | 0.427 | | | |
| **rs4986791** | | | | |
| C/C (n = 132) | 29 (22) | 29 (22) | 15 (11.4) | 59 (44.6) |
| C/T (n = 15) | 0 (0) | 3 (20) | 3 (20) | 9 (60) |
| p-value | 0.185 | | | |
| **rs2430561** | | | | |
| T/T (n = 69) | 17 (24.6) | 12 (17.4) | 7 (10.2) | 33 (47.8) |
| T/A (n = 66) | 12 (18.2) | 18 (27.3) | 6 (9.1) | 30 (45.4) |
| A/A (n = 13) | 0 (0) | 3 (23) | 5 (38.5) | 5 (38.5) |
| p-value | 0.038* | | | |
| **rs1800630** | | | | |
| C/C (n = 91) | 22 (24.2) | 20 (22) | 8 (8.8) | 41(45) |
| C/A (n = 50) | 6 (12) | 12 (24) | 10 (20) | 22 (44) |
| A/A (n = 7) | 1 (14.3) | 1 (14.3) | 0 (0) | 5 (71.4) |
| p-value | 0.231 | | | |

Db SNP ID: The Single Nucleotide Polymorphism Database identifier; rs: reference SNP; P: probability, p-values are unadjusted; * p < 0.05 indicates statistical significance.; n: number of individuals; %: frequencies ×100; SAC: South African Coloured.

**Supplementary** Table 7: The association between the investigated SNPs and periodontitis

across different smoking statuses.

| **Smoking status**  **SNPs Genotypes** | **Non Smokers**  **N (%)** | **Former Smokers**  **N (%)** | **Current Smokers**  **N (%)** |
| --- | --- | --- | --- |
| **rs1143634** | | | |
| GG (n = 105) | 66 (62.9) | 10 (9.5) | 29 (27.6) |
| GA (n = 37) | 21 (56.8) | 7 (18.9) | 9 (24.3) |
| AA (n = 4) | 3 (75) | 0 (0) | 1 (25) |
| p-value | 0.566 | | |
| **rs1800587** | | | |
| GG (n = 62) | 38 (61.3) | 7 (11.3) | 17 (27.4) |
| GA (n = 66) | 42 (63.7) | 9 (13.6) | 15 (22.7) |
| AA (n = 18) | 10 (55.6) | 1 (5.6) | 7 (38.8) |
| p-value | 0.670 | | |
| **rs17561** | | | |
| CC (n = 78) | 49 (62.8) | 7 (9) | 22 (28.2) |
| CA (n = 58) | 37 (63.8) | 9 (15.5) | 12 (20.7) |
| AA (n = 9) | 3 (33.3) | 1 (11.1) | 5 (55.6) |
| p-value | 0.193 | | |
| **rs419598** | | | |
| TT (n = 96) | 62 (64.6) | 10 (10.4) | 24 (25) |
| TC (n = 49) | 27 (55.1) | 7 (14.3) | 15 (30.6) |
| p-value | 0.530 | | |
| **rs1800629** | | | |
| G/G (n = 110) | 69 (60.5) | 14 (12.3) | 27 (23.7) |
| G/A (n = 35) | 21 (60) | 3 (8.6) | 11 (31.4) |
| A/A (n = 1) | 0 (0) | 0 (0) | 1 (100) |
| p-value | 0.454 | | |
| **rs361525** | | | |
| G/G (n = 125) | 76 (60.8) | 13 (10.4) | 36 (28.8) |
| G/A (n = 21) | 14 (66.7) | 4 (19) | 3 (14.3) |
| p-value | 0.263 | | |

| **Smoking status**  **SNPs Genotypes** | **Non Smokers**  **N (%)** | **Former Smokers**  **N (%)** | **Current Smokers**  **N (%)** |
| --- | --- | --- | --- |
| **rs2073618** | | | |
| C/C (n = 72) | 45 (62.5) | 8 (11.1) | 19 (26.4) |
| C/G (n = 57) | 35 (61.4) | 8 (14) | 14 (24.6) |
| G/G (n = 16) | 9 (56.3) | 1 (6.2) | 6 (37.5) |
| p-value | 0.815 | | |
| **rs1143627** | | | |
| G/G (n = 43) | 29 (67.4) | 4 (9.3) | 10 (23.3) |
| G/A (n = 68) | 39 (57.4) | 9 (13.2) | 20 (29.4) |
| A/A (n = 34) | 21 (61.8) | 4 (11.7) | 9 (26.5) |
| p-value | 0.885 | | |
| **rs16944** | | | |
| G/G (n = 43) | 27 (62.8) | 4 (9.3) | 12 (27.9) |
| G/A (n = 66) | 37 (56) | 10 (15.2) | 19 (28.8) |
| A/A (n = 37) | 26 (70.3) | 3 (8.1) | 8 (21.6) |
| p-value | 0.631 | | |
| **rs1800795** | | | |
| G/G (n = 95) | 61 (64.2) | 12 (12.6) | 22 (23.2) |
| G/C (n = 41) | 25 (61) | 4 (9.8) | 12 (29.2) |
| C/C (n = 9) | 4 (44.4) | 0 (0) | 5 (55.6) |
| p-value | 0.274 | | |
| **rs1800872** | | | |
| G/G (n = 72) | 37 (51.4) | 12 (16.7) | 23 (31.9) |
| G/T (n = 60) | 44 (73.3) | 5 (8.3) | 11 (18.4) |
| T/T (n = 14) | 9 (64.3) | 0 (0) | 5 (35.7) |
| p-value | 0.059 | | |
| **rs1800871** | | | |
| G/G (n = 72) | 37 (51.4) | 12 (16.7) | 23 (31.9) |
| G/A (n = 59) | 43 (72.9) | 5 (8.5) | 11 (18.6) |
| A/A (n = 14) | 9 (64.3) | 0 (0) | 5 (35.7) |
| p-value | 0.069 | | |
| **rs1800896** | | | |
| T/T (n = 56) | 39 (69.6) | 6 (10.7) | 11 (19.6) |
| T/C (n = 70) | 41 (58.6) | 9 (12.9) | 20 (28.5) |
| C/C (n = 19) | 9 (47.4) | 2 (10.5) | 8 (42.1) |
| p-value | 0.370 | | |

| **Smoking status**  **SNPs Genotypes** | **Non Smokers**  **N (%)** | **Former Smokers**  **N (%)** | **Current Smokers**  **N (%)** |
| --- | --- | --- | --- |
| **rs11225395** | | | |
| G/G (n = 62) | 39 (62.9) | 7 (11.3) | 16 (25.8) |
| G/A (n = 70) | 42 (60) | 7 (10) | 21 (30) |
| A/A (n = 14) | 9 (64.3) | 3 (21.4) | 2 (14.2) |
| p-value | 0.646 | | |
| **rs2277438** | | | |
| A/A (n = 105) | 66 (62.9) | 12 (11.4) | 27 (25.7) |
| A/G (n = 36) | 19 (52.8) | 5 (13.9) | 12 (33.3) |
| G/G (n = 5) | 5 (100) | 0 (0) | 0 (0) |
| p-value | 0.356 | | |
| **rs35211496** | | | |
| C/C (n = 128) | 77 (60.2) | 16 (12.5) | 35 (27.3) |
| C/T (n = 17) | 12 (70.6) | 1 (5.9) | 4 (23.5) |
| T/T (n = 1) | 1 (100) | 0 (0) | 0 (0) |
| p-value | 0.820 | | |
| **rs2243250** | | | |
| C/C (n = 50) | 29 (58) | 6 (12) | 15 (30) |
| C/T (n = 61) | 36 (59) | 8 (13) | 17 (28) |
| T/T (n = 35) | 25 (71.4) | 3 (8.6) | 7 (20) |
| p-value | 0.746 | | |
| **rs2070874** | | | |
| C/C (n = 59) | 35 (59.3) | 8 (13.6) | 16 (27.1) |
| C/T (n = 63) | 38 (60.3) | 6 (9.5) | 19 (30.2) |
| T/T (n = 24) | 17 (70.8) | 3 (12.5) | 4 (16.7) |
| p-value | 0.730 | | |
| **rs2275913** | | | |
| G/G (n = 75) | 48 (64) | 10 (13.3) | 17 (22.7) |
| G/A (n = 60) | 34 (56.7) | 6 (10) | 20 (33.3) |
| A/A (n = 10) | 7 (70) | 1 (10) | 2 (20) |
| p-value | 0.669 | | |
| **rs4986790** | | | |
| A/A (n = 132) | 83 (62.9) | 15 (11.4) | 34 (25.7) |
| A/G (n = 13) | 6 (46.2) | 2 (15.4) | 5 (38.4) |
| p-value | 0.493 | | |

| **Smoking status**  **SNPs Genotypes** | **Non Smokers**  **N (%)** | **Former Smokers**  **N (%)** | **Current Smokers**  **N (%)** |
| --- | --- | --- | --- |
| **rs4986791** | | | |
| C/C (n = 131) | 83 (63.3) | 15 (11.5) | 33 (25.2) |
| C/T (n = 14) | 6 (42.9) | 2 (14.2) | 6 (42.9) |
| p-value | 0.298 | | |
| **rs2430561** | | | |
| T/T (n = 68) | 44 (64.7) | 6 (8.8) | 18 (26.5) |
| T/A (n = 65) | 39 (60) | 10 (15.4) | 16 (24.6) |
| A/A (n = 13) | 7 (30.8) | 1 (7.7) | 5 (38.5) |
| p-value | 0.652 | | |
| **rs1800630** | | | |
| C/C (n = 91) | 56 (61.5) | 9 (9.9) | 26 (28.6) |
| C/A (n = 49) | 31 (63.3) | 6 (12.2) | 12 (24.5) |
| A/A (n = 6) | 3 (50) | 2 (33.3) | 1 (16.7) |
| p-value | 0.514 | | |

db SNP ID: The Single Nucleotide Polymorphism Database identifier; rs: reference SNP; P: probability, p-values are unadjusted; * p < 0.05 indicates statistical significance; n: number of individuals; %: frequencies ×100.

Supplementary Table 8: Correlation of samples analysed for bacterial profiles

with their corresepomding genetic profile.

| **SNPs** | **dbSNP**  **ID** | **Case**  **1** | **Case**  **3** | **Case**  **4** | **Case**  **5** | **Case**  **7** | **Prevalent of genotype**  **(%)** |  | **Control**  **2** | **Control**  **4** | **Control**  **9** | **Control**  **12** | **Control**  **13** | **Prevalent of genotype**  **(%)** |
| --- | --- | --- | --- | --- | --- | --- | --- | --- | --- | --- | --- | --- | --- | --- |
| **IL-1B**  **+3954/3953** | **rs1143634** | GA | GA | GG | GG | GG | GG=60 |  | GG | GG | GG | GG | GG | GG=100 |
|  |  |  |  |  |  |  | GA=40 |  |  |  |  |  |  | GA=0 |
|  |  |  |  |  |  |  | AA=0 |  |  |  |  |  |  | AA=0 |
| **IL-1A**  **-889** | **rs1800587** | AA | GA | GG | GG | GG | GG=60 |  | GA | GG | GA | GG | GG | GG=60 |
|  |  |  |  |  |  |  | GA=20 |  |  |  |  |  |  | GA=40 |
|  |  |  |  |  |  |  | GG=20 |  |  |  |  |  |  | AA=0 |
| **IL-1A +4845** | **rs17561** | AC | AC | CC | CC | CC | CC=60 |  | CC | CC | AC | CC | CC | CC=80 |
|  |  |  |  |  |  |  | AC=40 |  |  |  |  |  |  | AC=20 |
|  |  |  |  |  |  |  | AA=0 |  |  |  |  |  |  | AA=0 |
| **IL-1RN +2018** | **rs419598** | TT | TT | CT | TT | TT | TT=80 |  | TT | CT | TT | TT | TT | TT=80 |
|  |  |  |  |  |  |  | CT=20 |  |  |  |  |  |  | CT=20 |
|  |  |  |  |  |  |  | CC=0 |  |  |  |  |  |  | CC=0 |
| **TNF-α**  **-308** | **rs1800629** | AG | GG | GG | GG | AG | GG=60 |  | GG | GG | GG | GG | GA | GG=80 |
|  |  |  |  |  |  |  | AG=40 |  |  |  |  |  |  | GA=20 |
|  |  |  |  |  |  |  | AA=0 |  |  |  |  |  |  | AA=0 |
| **TNF-α**  **-238** | **rs361525** | **GG** | AG | **GG** | AG | **GG** | GG=60 |  | **GG** | **GG** | AG | **GG** | **GG** | GG=80 |
|  |  |  |  |  |  |  | AG=40 |  |  |  |  |  |  | AG=20 |
|  |  |  |  |  |  |  | AA=0 |  |  |  |  |  |  | AA=0 |
| **OPG**  **+1181** | **rs2073618** | **CC** | **CC** | **CC** | **CC** | **CC** | CC=100 |  | **GG** | **CC** | **CC** | **CC** | GC | CC=60 |
|  |  |  |  |  |  |  | GC=0 |  |  |  |  |  |  | CG=20 |
|  |  |  |  |  |  |  | GG=0 |  |  |  |  |  |  | GG=20 |
| **IL-1B**  **-31** | **rs1143627** | AA | AA | GG | GA | AA | AA=60 |  | GA | GA | GG | GG | AA | GG=40 |
|  |  |  |  |  |  |  | GA=20 |  |  |  |  |  |  | GA=40 |
|  |  |  |  |  |  |  | GG=20 |  |  |  |  |  |  | AA=20 |

db SNP ID: The Single Nucleotide Polymorphism Database identifier; rs: reference SNP; n: number of individuals.

(continued): Correlation of samples analysed for bacterial profiles

with their corresepomding genetic profile.

| **SNPs** | **dbSNP**  **ID** | **Case**  **1** | **Case**  **3** | **Case**  **4** | **Case**  **5** | **Case**  **7** | **Prevalent of genotype**  **(%)** |  | **Control**  **2** | **Control**  **4** | **Control**  **9** | **Control**  **12** | **Control**  **13** | **Prevalent of genotype**  **(%)** |
| --- | --- | --- | --- | --- | --- | --- | --- | --- | --- | --- | --- | --- | --- | --- |
| **IL-1B**  **-511** | **rs16944** | **GG** | **GG** | AA | **GG** | **GG** | GG=80 |  | **GG** | GA | AA | **GG** | **GG** | GG=60 |
|  |  |  |  |  |  |  | GA=0 |  |  |  |  |  |  | GA=20 |
|  |  |  |  |  |  |  | AA=20 |  |  |  |  |  |  | AA=20 |
| **IL 6**  **-174** | **rs1800795** | GG | GG | GG | GG | CC | GG=80 |  | GG | GC | GG | GC | GC | GG=60 |
|  |  |  |  |  |  |  | GC=0 |  |  |  |  |  |  | GC=40 |
|  |  |  |  |  |  |  | CC=20 |  |  |  |  |  |  | CC=0 |
| **IL-10**  **-592** | **rs1800872** | GG | TG | TG | TG | GG | GG=40 |  | TT | TG | TG | TG | TG | TT=20 |
|  |  |  |  |  |  |  | TG=60 |  |  |  |  |  |  | TG=80 |
|  |  |  |  |  |  |  | TT=0 |  |  |  |  |  |  | GG=0 |
| **IL-10**  **-819** | **rs1800871** | GG | AG | AG | AG | GG | GG=40 |  | AA | AG | AG | AG | AG | AA=20 |
|  |  |  |  |  |  |  | GA=60 |  |  |  |  |  |  | AG=80 |
|  |  |  |  |  |  |  | AA=0 |  |  |  |  |  |  | GG=0 |
| **IL 10**  **-1082** | **rs1800896** | TC | TC | TC | TT | TC | TT=20 |  | TT | TT | TC | TT | TC | TT=60 |
|  |  |  |  |  |  |  | TC=80 |  |  |  |  |  |  | TC=40 |
|  |  |  |  |  |  |  | CC=0 |  |  |  |  |  |  | CC=0 |
| **MMP-8**  **-799** | **rs11225395** | GG | GG | GG | GG | AG | GG=80 |  | AG | AG | AG | GG | AG | GG=20 |
|  |  |  |  |  |  |  | AG=20 |  |  |  |  |  |  | AG=80 |
|  |  |  |  |  |  |  | AA=0 |  |  |  |  |  |  | AA=0 |
| **RANKL RL2** | **rs2277438** | GA | GA | **AA** | **AA** | **AA** | AA=60 |  | GA | **AA** | **AA** | **AA** | **AA** | AA=80 |
|  |  |  |  |  |  |  | GA=40 |  |  |  |  |  |  | GA=20 |
|  |  |  |  |  |  |  | GG=0 |  |  |  |  |  |  | GG=0 |
| **RANK**  **RK** | **rs35211496** | CC | CC | CC | CC | CT | CC=80 |  | CC | CC | CC | CC | CC | CC=100 |
|  |  |  |  |  |  |  | CT=20 |  |  |  |  |  |  | CT=0 |
|  |  |  |  |  |  |  | TT=0 |  |  |  |  |  |  | TT=0 |

db SNP ID: The Single Nucleotide Polymorphism Database identifier; rs: reference SNP; n: number of individuals.

(continued): Correlation of samples analysed for bacterial profiles

with their corresepomding genetic profile.

| **SNPs** | **dbSNP**  **ID** | **Case**  **1** | **Case**  **3** | **Case**  **4** | **Case**  **5** | **Case**  **7** | **Prevalent of genotype**  **(%)** |  | **Control**  **2** | **Control**  **4** | **Control**  **9** | **Control**  **12** | **Control**  **13** | **Prevalent of genotype**  **(%)** |
| --- | --- | --- | --- | --- | --- | --- | --- | --- | --- | --- | --- | --- | --- | --- |
| **IL-4**  **-590** | **rs2243250** | CT | CC | CC | TT | CT | CC=60 |  | CC | CT | CC | CC | CT | CC=60 |
|  |  |  |  |  |  |  | CT=40 |  |  |  |  |  |  | CT=40 |
|  |  |  |  |  |  |  | TT=0 |  |  |  |  |  |  | TT=0 |
| **IL-4**  **-33** | **rs2070874** | CC | CC | CT | CT | CC | CC=60 |  | CT | CT | CC | CC | CT | CC=60 |
|  |  |  |  |  |  |  | CT=40 |  |  |  |  |  |  | CT=40 |
|  |  |  |  |  |  |  | TT=0 |  |  |  |  |  |  | TT=0 |
| **IL-17A**  **+197** | **rs2275913** | AG | AG | **GG** | **GG** | **GG** | GG=60 |  | AA | AG | **GG** | AG | AG | GG=20 |
|  |  |  |  |  |  |  | AG=40 |  |  |  |  |  |  | AG=60 |
|  |  |  |  |  |  |  | AA=0 |  |  |  |  |  |  | AA=20 |
| **TLR-4**  **+896** | **rs4986790** | AA | AA | AA | AA | AA | AA=100 |  | AA | AG | AA | AA | AA | AA=80 |
|  |  |  |  |  |  |  | AG=0 |  |  |  |  |  |  | AG=20 |
|  |  |  |  |  |  |  | GG=0 |  |  |  |  |  |  | GG=0 |
| **TLR-4**  **+1196** | **rs4986791** | CC | CC | CC | CC | CC | CC=100 |  | CC | CT | CC | CC | CC | CC=80 |
|  |  |  |  |  |  |  | CT=0 |  |  |  |  |  |  | CT=20 |
|  |  |  |  |  |  |  | TT=0 |  |  |  |  |  |  | TT=0 |
| **IFN-𝛾**  **+874** | **rs2430561** | TA | TT | TT | TT | TA | TT=60 |  | TA | TA | TA | TA | TT | TT=20 |
|  |  |  |  |  |  |  | TA=40 |  |  |  |  |  |  | TA=80 |
|  |  |  |  |  |  |  | AA=0 |  |  |  |  |  |  | AA=0 |
| **TNF-α**  **-863** | **rs1800630** | CC | CC | CC | CA | CC | CC=80 |  | CA | CA | CC | CC | CC | CC=60 |
|  |  |  |  |  |  |  | CA=20 |  |  |  |  |  |  | CA=40 |
|  |  |  |  |  |  |  | AA=0 |  |  |  |  |  |  | AA=0 |

db SNP ID: The Single Nucleotide Polymorphism Database identifier; rs: reference SNP; n: number of individuls.

Supplementary Figure 1: Participant Flow Diagram**.** Flow of participants through recruitment, eligibility assessment, genotyping, and final analysis. All 150 participants completed full assessments, and genotyping success exceeded 95%. One SNP (IL-6 −572, rs1800796) was excluded for deviation from Hardy–Weinberg equilibrium.


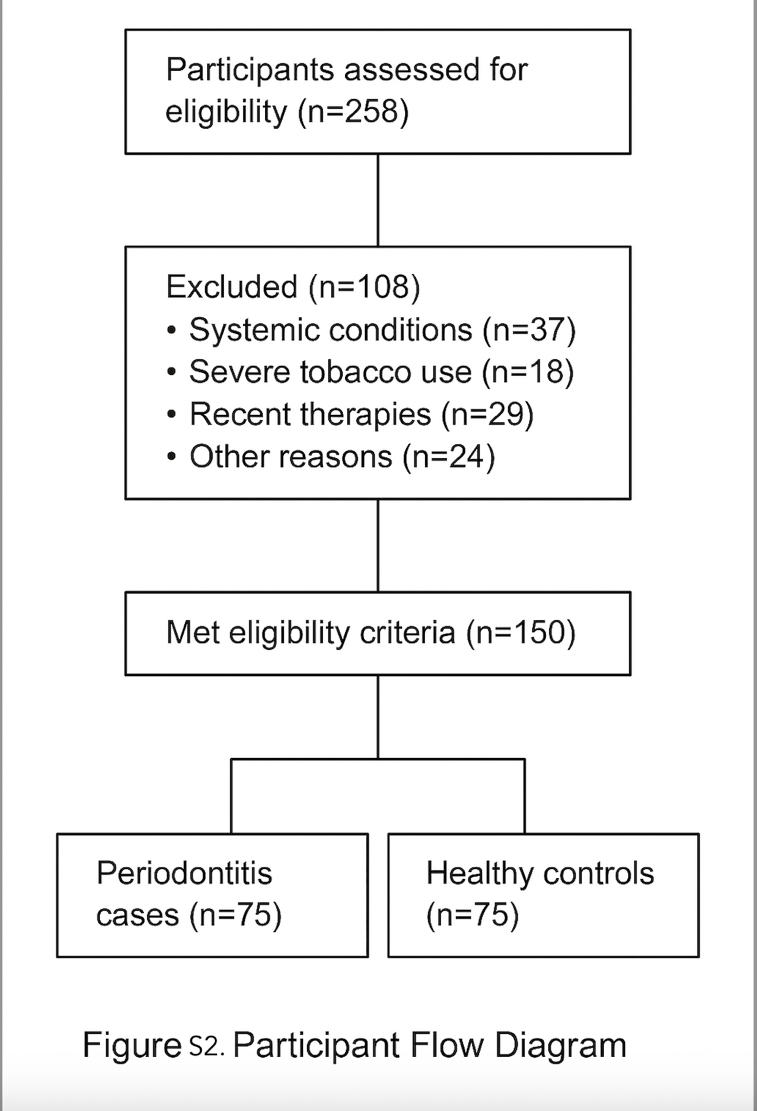


Supplementary Figure 2: STRUCTURE bar plot showing population stratification at K = 2. Each vertical bar represents one individual, and colors indicate estimated membership proportions in the two inferred genetic clusters (Cluster 1 = blue, Cluster 2 = orange). The even distribution and mixed membership patterns reflect moderate admixture within the study population.
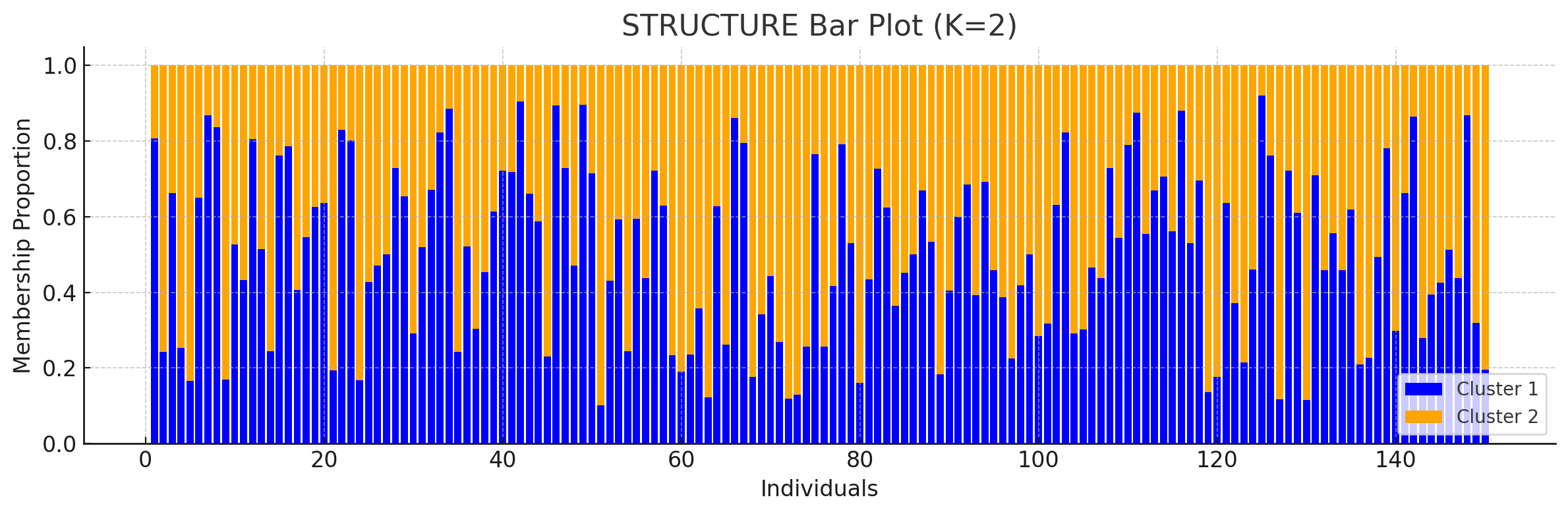

Supplement: Supplementary file 1 [file mmc1.docx]
